# Efficacy of intrathecal mesenchymal stem cell-neural progenitor therapy in progressive MS: results from a phase II, randomized, placebo-controlled clinical trial

**DOI:** 10.1186/s13287-024-03765-6

**Published:** 2024-05-23

**Authors:** Violaine K. Harris, James Stark, Armistead Williams, Morgan Roche, Michaela Malin, Anjali Kumar, Alyssa L. Carlson, Cara Kizilbash, Jaina Wollowitz, Caroline Andy, Linda M. Gerber, Saud A. Sadiq

**Affiliations:** 1 Tisch Multiple Sclerosis Research Center of New York, New York, NY 10019 USA; 2 Weill Cornell Medicine, Department of Population Health Sciences, New York, NY USA

**Keywords:** Multiple sclerosis, Clinical trial, Mesenchymal stem cell, MSC-NP, Intrathecal, Cell therapy, Progressive multiple sclerosis

## Abstract

**Background:**

Mesenchymal stem cell-neural progenitors (MSC-NPs) are a bone marrow mesenchymal stem cell (MSC)-derived ex vivo manipulated cell product with therapeutic potential in multiple sclerosis (MS). The objective of this study was to determine efficacy of intrathecal (IT) MSC-NP treatment in patients with progressive MS.

**Methods:**

The study is a phase II randomized, double-blind, placebo-controlled clinical trial with a compassionate crossover design conducted at a single site. Subjects were stratified according to baseline Expanded Disability Status Scale (EDSS) (3.0-6.5) and disease subtype (secondary or primary progressive MS) and randomized into either treatment or placebo group to receive six IT injections of autologous MSC-NPs or saline every two months. The primary outcome was EDSS Plus, defined by improvement in EDSS, timed 25-foot walk (T25FW) or nine-hole peg test. Secondary outcomes included the individual components of EDSS Plus, the six-minute walk test (6MWT), urodynamics testing, and brain atrophy measurement.

**Results:**

Subjects were randomized into MSC-NP (*n* = 27) or saline (*n* = 27) groups. There was no difference in EDSS Plus improvement between the MSC-NP (33%) and saline (37%) groups. Exploratory subgroup analysis demonstrated that in subjects who require assistance for ambulation (EDSS 6.0-6.5) there was a significantly higher percentage of improvement in T25FW and 6MWT in the MSC-NP group (3.7% ± 23.1% and − 9.2% ± 18.2%) compared to the saline group (-54.4% ± 70.5% and − 32.1% ± 30.0%), (*p* = 0.030 and *p* = 0.036, respectively). IT-MSC-NP treatment was also associated with improved bladder function and reduced rate of grey matter atrophy on brain MRI. Biomarker analysis demonstrated increased MMP9 and decreased CCL2 levels in the cerebrospinal fluid following treatment.

**Conclusion:**

Results from exploratory outcomes suggest that IT-MSC-NP treatment may be associated with a therapeutic response in a subgroup of MS patients.

**Trial Registration:**

ClinicalTrials.gov NCT03355365, registered November 14, 2017, https://clinicaltrials.gov/study/NCT03355365?term=NCT03355365&rank=1.

**Supplementary Information:**

The online version contains supplementary material available at 10.1186/s13287-024-03765-6.

## Introduction

Multiple sclerosis (MS) is a chronic inflammatory disease of the central nervous system (CNS) affecting approximately 2.8 million people worldwide [1]. Physical disability in MS is attributed to a characteristic pathology consisting of discrete white matter lesions of acute inflammatory demyelination and reactive astrogliosis [2]. Cortical gray matter demyelination and brain atrophy are additional pathological features of the disease [3–5]. The clinical symptoms of MS typically manifest in young adulthood as relapses of neurological dysfunction that resolve with variable periods of remission. Over time, the relapsing remitting course of MS (RRMS) and the accompanying accumulation of lesion burden and neuronal degeneration may evolve into secondary progressive MS (SPMS) associated with progressive clinical disability. Approximately 10–15% of usually older patients experience progressive disability from symptom onset and are diagnosed with primary progressive MS (PPMS) [6]. The predominant inflammatory demyelination observed in early disease can lead to accumulating axonal loss and neurodegeneration resulting in irreversible neurological disability in progressive disease [4]. Most of the currently approved therapies for MS target immune mechanisms and exhibit varying degrees of efficacy in treating active RRMS [7]. However, patients with SPMS or PPMS with established disability in the absence of ongoing relapses or active lesions on MRI have no viable therapeutic options since current therapies are ineffective [8].

We investigated the use of autologous bone marrow mesenchymal stem cell (MSC)-derived neural progenitors (MSC-NPs) as a therapeutic option for MS patients with progressive disease. MSC-NPs are a derivative of MSCs with enriched expression of neural and cell signaling genes, and a reduced capacity to differentiate into mesodermal cell types [9, 10]. MSC-NPs exhibit many of the immunomodulatory and trophic properties associated with MSC-mediated tissue repair, including suppression of microglial activation via paracrine signaling [9, 11]. Importantly, the equivalence of MSCs derived from MS compared to non-MS or healthy donors was demonstrated with respect to growth rate in culture, suppression of T-cell proliferation, promotion of oligodendrocyte differentiation, and transcriptomic signature, supporting the use of autologous MSCs in MS [9, 10]. The potential for therapeutic efficacy was first shown in the murine model of MS, where multiple intrathecal (IT) injections of MSC-NPs reduced neurological symptoms and improved pathology including increased spinal cord myelination, decreased immune infiltration in the CNS, and increased recruitment of endogenous neural progenitor cells [12]. Although MSC-NPs exhibit a neural phenotype and upregulate neural genes in vitro, differentiation into neural lineages was not observed following transplantation in vivo, consistent with a therapeutic mechanism based on paracrine signaling through the release of trophic and immunomodulatory factors [10, 12].

The safety and tolerability of the multiple dosing regimen of IT MSC-NPs was previously established in a small pilot study followed by an open-label phase I trial in 20 MS patients with advanced progressive disease [13–15]. Importantly, these early clinical studies demonstrated positive trends in walking outcomes, muscle strength and bladder function indicating possible efficacy in reducing disability [13, 15]. A 2-year follow-up study of the phase I cohort demonstrated sustained benefit in a subset of patients and a lack of long-term adverse effects [14].

The current study is a phase II, randomized, double-blind, placebo-controlled clinical trial with a compassionate crossover design. The aim of the study is to determine efficacy of six IT injections of autologous bone marrow-derived MSC-NPs in ambulatory, non-relapsing SPMS and PPMS patients who lack MRI evidence of active disease.

## Methods

### Study design and oversight

The study is an investigator-initiated, phase II, double-blind, placebo-controlled, randomized, parallel group clinical trial with a compassionate crossover design. All study activities were conducted at the Tisch MS Research Center of New York, USA. The study was conducted as an FDA investigational new drug, and is registered with ClinicalTrials.gov, number NCT03355365. The ethics of the study was approved on 11/28/2016 by Western Institutional Review Board (reference number 20,162,572). All patients gave written informed consent. An independent external data and safety monitoring board evaluated all safety data on a yearly basis.

### Participants

Eligible patients had clinically definite SPMS or PPMS with EDSS disability score between 3.0 and 6.5 with stable disease as determined by less than a 1.0 point change in EDSS and an absence of clinical relapses in the 12 months prior to enrollment, and by lack of gadolinium-enhancing lesions with stable MRI disease burden (number and size of T2 lesions) in the prior six months. Enrollment criteria included disease duration of less than 20 years at time of screening (based on onset of symptoms when symptom onset was clearly defined or based on date of diagnosis if symptom onset was difficult to determine); no change of disease modifying therapy (DMT) less than 12 months prior to beginning the trial; and no change in MS symptom medications, including dalfampridine less than six months prior to study treatment. Subjects with existing medical comorbidities or cancer history that might complicate safety outcomes of the experimental treatment were excluded.

### Randomization and masking

Eligible subjects were stratified by disease subtype (SPMS or PPMS) and baseline EDSS score (3.0–4.0, 4.5–5.5, 6.0, and 6.5). The study aimed to enroll a total of 50 subjects, including 40 SPMS subjects distributed in each of the 4 EDSS blocks (10 subjects per block), and 10 PPMS subjects with four in EDSS block 3.0–4.0 and two each in EDSS blocks 4.5–5.5, 6.0, and 6.5. After ensuring eligibility criteria, subjects in each stratum were block randomized into either the saline or MSC-NP treatment group in order of date of consent based on a pre-determined randomization scheme generated by LG who was not involved in patient enrollment or treatment. Subject allocation and coordination of cell manufacturing was performed by VH, who was unblinded. The single procedure neurologist (SS) who performed all lumbar punctures and intrathecal injection procedures was also unblinded and did not perform any clinical or safety assessments on study participants. All study participants, neurologists assessing clinical outcomes or adverse events, and clinical study coordinators involved in enrollment and data collection were blinded to the treatment assignment.

### Procedures

Cell manufacturing was performed in a cGMP facility at the Tisch MS Research Center of New York. Detailed methods of MSC and MSC-NP manufacturing and release criteria have been described previously and are included in the supplementary material (Supplemental Methods) [9, 13]. In brief, MSCs were isolated from a single bone marrow aspirate from each subject, expanded ex vivo, and cryopreserved at passages 2 and 3. Prior to each treatment, a portion of cryopreserved MSCs were thawed, expanded for 2 more passages, and cultured for 2 weeks in neural maintenance media to generate MSC-NPs. All individual batches of MSC-NPs conformed to release criteria as detailed in the Supplemental Methods. To preserve functionality of the cells, final product autologous MSC-NPs were collected directly from cell culture, washed, and cell count/viability was determined just prior to each injection. Viability was > 80% for all batches of MSC-NPs. A dose of up to 10 million cells was resuspended in preservative-free saline, transferred to an unlabeled sterile tube and immediately transported to the procedure neurologist in a container with a pre-specified label. Placebo samples consisted of an empty tube transported in an identical container with a pre-specified placebo label.

In year one of the study, subjects assigned to the MSC-NP group received six separate IT injections of up to 1 × 10^7^ autologous MSC-NPs spaced two months apart (treatments one through six). Subjects in the placebo group received IT injections of saline following the same schedule. Lumbar puncture and CSF aspiration was performed as previously described [13]. The cell suspension was removed from the transport vial using a 22-gauge needle 10 cc syringe and diluted in 3 ml of preservative-free sterile saline before IT injection. Injection was followed by a 2 ml saline flush. For placebo subjects, the entire procedure was performed with saline only. In two of the subjects, cells were administered intrathecally via an in-dwelling access port of an implanted baclofen pump. All procedures took place posterior to the study subject and behind a screen to ensure that the patient and assisting nurse remained blinded to the treatment. Following each procedure, subjects were placed in the Trendelenburg position for one hour. Prophylactic IV infusion of antibiotics (80 mg of tobramycin and 500 mg of vancomycin) was co-administered, and prophylactic oral acetaminophen was administered to minimize headaches.

The experimental design included a compassionate crossover element for purposes of masking (ensuring all subjects had a bone marrow procedure) and compassionate use of the MSC-NP treatment for subjects receiving placebo. Therefore, in year two of the study, placebo subjects crossed over into the MSC-NP treatment group and received six separate IT injections of autologous MSC-NPs as part of a compassionate crossover design (treatments seven through 12). Similarly, subjects receiving MSC-NPs in year one received saline injections in year two.

### Outcomes

The primary outcome was EDSS Plus, defined as improvement in either EDSS, timed 25-ft walk (T25FW), or nine-hole peg test (9HPT) [16]. Improvement was defined by at least one of the following three pre-specified measures: ≥0.5 improvement in EDSS (if EDSS at entry is ≥ 6.0) or ≥ 1.0 improvement in EDSS (if EDSS at entry is ≤ 5.5), ≥ 20% improvement in T25FW, and ≥ 20% improvement in 9HPT in either dominant or non-dominant upper limb. Secondary outcome measures included EDSS, T25FW, 9HPT, six-minute walk test (6MWT), 12-item walking scale (MSWS-12), multiple sclerosis functional composite (MSFC), paced auditory serial addition test (PASAT), urodynamics and MRI imaging. T25FW and 9HPT were analyzed as both a continuous measure (% change after one year) and a binary measure (≥ 20% improvement or ≥ 20% worsening compared to baseline) based on previously defined cutoffs determined to be clinically meaningful [16]. All outcome assessments were performed at baseline, two months after the sixth treatment and two months after the 12th treatment. Additional EDSS Plus assessments (EDSS, T25FW and 9HPT) were performed at mid-year timepoints (treatments four and ten).

To assess bladder function, a history of symptoms was taken and any use of medications affecting bladder function was noted. Subjects underwent urodynamic testing performed in the same laboratory and results interpreted by a single neuro-urologist. Bladder function measurements included post-void residual volume (PVR) and maximum bladder capacity. Bladder function improvement was determined by a decrease in PVR of > 20% after 1 year of treatment compared to baseline.

Brain, cervical and thoracic spine MRI scans with and without gadolinium enhancement were read by a neuro-radiologist. As an exploratory outcome, images were analyzed using the NeuroQuant® volumetric MRI software (CorTechs Labs, Inc.; La Jolla, California, USA) which measures and compares volumes of brain structures to a normative database adjusted for age, gender and intracranial volume. A multi-structure atrophy report was generated for all brain MRI scans as well as a LesionQuant™ Flair assessment of total number and volume of lesions.

Each subject was assigned to an assessing neurologist who performed and oversaw all outcome assessments, and a separate adverse event (AE) neurologist to whom all AEs were reported. All subjects were contacted two, seven, and 30 days following each procedure and asked if they experienced any AEs including headaches. All AEs were reported and graded following the NCI Common Terminology Criteria for Adverse Events (CTCAE version 4.0). If a subject reported a headache, they completed the headache pain scale (0–10 scale). Headaches were categorized as mild (0–3), moderate (4–7) or severe (8–10). The clinical data collection and management was performed using the OpenClinica open-source software, version 3.13.1.

### CSF biomarker analysis

As part of the study protocol, CSF was collected at each IT procedure just prior to cell or saline injection. Cell-free CSF was processed as previously described [17]. For biomarker discovery, two CSF samples representing baseline and post-MSC-NP treatment from 36 subjects were included for a total of 72 samples. For the majority of sample pairs (26/36), CSF obtained at the time of the first MSC-NP treatment was used as a baseline sample, and CSF obtained at the time of the sixth MSC-NP treatment (reflecting five treatments) was used as a post-treatment sample. In the remaining pairs (10/36) the timing of baseline or post-treatment samples was modified to avoid blood contaminated samples. Twenty subjects received MSC-NP treatments in year one, and 16 subjects received MSC-NP treatments in year two.

Proteomic analysis of CSF was performed using Slow Off-rate Modified Aptamers assay (SOMAScan Assay v4.1, SomaLogic, Inc). SOMAmer-protein binding was quantified using DNA-hybridization microarrays and normalized using hybridization controls. Median signal normalization was performed using Adaptive Normalization by Maximum Likelihood. On average, 17.2% of SOMAmers fell below the limit of detection (range 2.3–46.4%), suggesting low signal in CSF samples. Signal was log2 transformed for statistical analysis. Candidate biomarkers were validated in CSF samples from all trial subjects who received MSC-NP treatment. MMP9 and CCL2 were measured in undiluted CSF using human MMP9 magnetic Luminex performance assay (R&D Systems) and Bio-Plex Pro human cytokine panel from Bio-Rad, respectively. Bio-Plex Pro 200 system (Bio-Rad) was used for analyte detection. Responders to MSC-NP treatment were defined by improvement in any of the following outcomes following MSC-NP treatment: EDSS (≥ 0.5 point improvement), timed 25-foot walk (≥ 20% improvement), or 9-hole peg test (≥ 20% improvement). Non-responders were defined by stability or worsening in any of the outcomes.

### Statistical analysis

Sample size of 20 in the treatment group and 20 in the placebo group was calculated to achieve more than 80% power to detect a difference between the group proportions of 35%, using a two-sided Z-test with pooled variance and using a significance level of 0.05. This calculation assumes that 40% of patients in the treatment group and 5% of patients in the placebo group achieve at least one of the EDSS-Plus components. The prevalence of patients with improved outcome was informed by the results of the phase I trial, in which 40% of patients experienced a decrease of 0.5 or more on EDSS [13]. Target enrollment of 50 patients (25 patients in each group) was designed to account for an expected attrition rate of 20%.

The primary outcome, EDSS Plus improvement, and secondary outcomes were analyzed by treatment in the first year. Counts and percentages are displayed for categorical variables (EDSS Plus improvement, T25FW improvement, and T25FW worsening) and median and interquartile range for continuous variables (percent change in EDSS Plus, T25FW, 6MWT, 9HPT in the dominant hand, 9HPT in the non-dominant hand, MSFC, MSWS-12 and PASAT). Univariate hypothesis tests (Mann-Whitney U test for continuous variables, Chi squared tests or Fisher’s exact tests for categorical variables) were performed to compare each variable between placebo and MSC-NP treated patients using R version 4.2.3. The T25FW and 6MWT walking outcomes were then reassessed within low (3.0-5.5) and high (6.0-6.5) EDSS strata.

Differentially expressed proteins were identified from proteomic analysis using a multivariate linear mixed effect model with patient age and EDSS modeled as a continuous covariate and other independent variables (treatment, diagnosis, and gender) as discrete. The model was performed using R version 4.0.3. Pairwise contrasts (post-treatment minus pre-treatment) were extracted with least squares means function and candidate biomarkers selected based on unadjusted p-values (*p* < 0.01). Differences between biomarker levels before and after treatment were analyzed using Wilcoxon matched-pairs signed rank test, and significance of longitudinal values was determined by Friedman test followed by Dunn’s multiple comparisons test using GraphPad Prism 9. Differences in PVR volumes and grey matter volumes were determined by Mann-Whitney test.

## Results

**Fig. 1:**
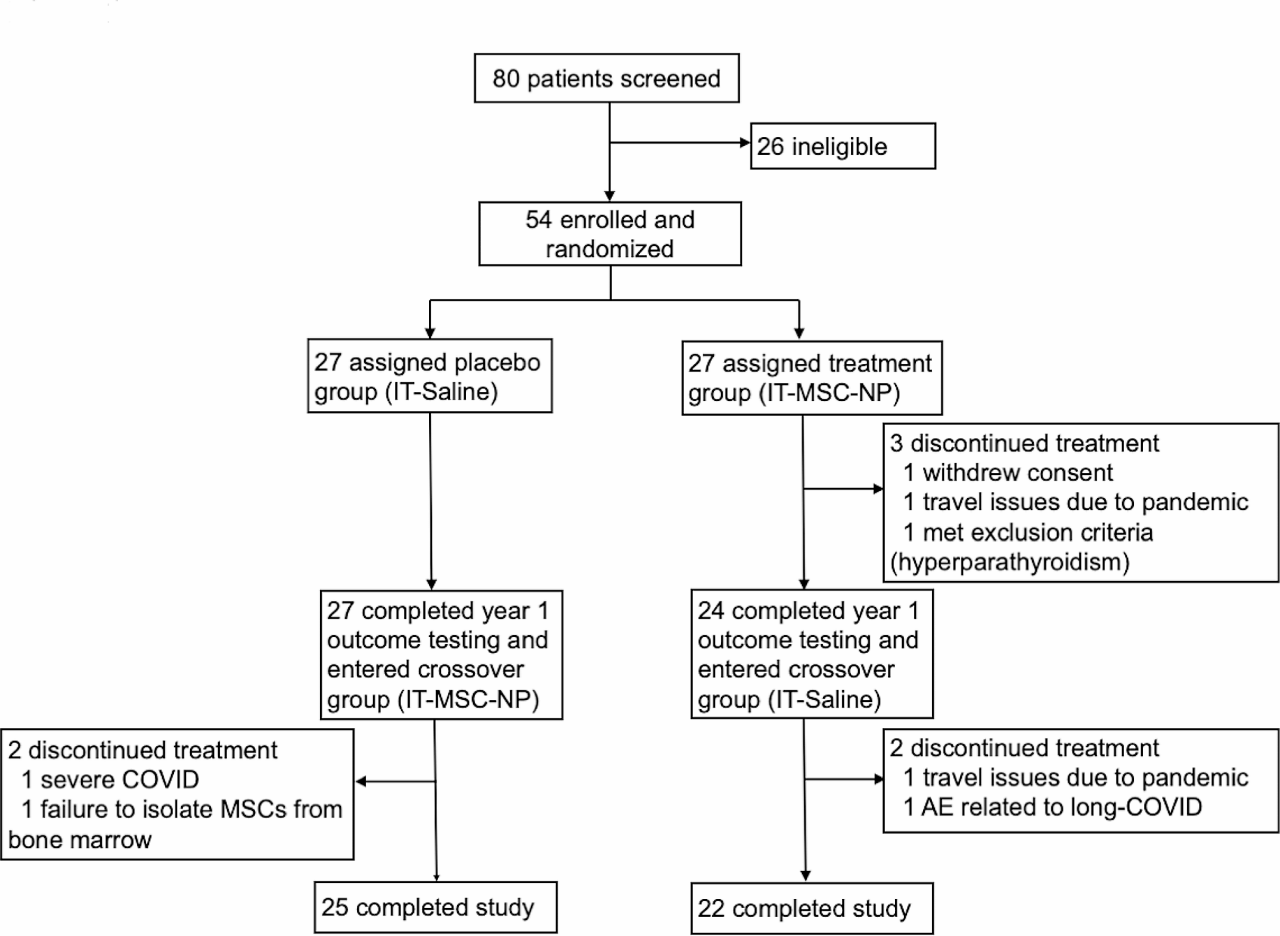
Trial design and enrollment IT, intrathecal; MSC-NP, mesenchymal stem cell-derived neural progenitor

Study participants with SPMS or PPMS were enrolled between August 10, 2018 and June 2, 2020. Of the 80 patients screened, 54 were enrolled and randomized, and 51 completed all treatments and outcome assessments for year one (Fig. 1). Upon enrollment, subjects were stratified into blocks according to disease subtype and EDSS and randomized into IT-MSC-NP or IT-saline groups within each block (Table 1). The two groups showed similar demographics and baseline characteristics in terms of sex, age, and disease duration (Table 1). Subjects received concomitant DMTs as detailed in Table 1, with the majority (35/51, 69%) receiving concomitant anti-CD20 therapy (ocrelizumab/rituximab).

**Table 1:** Subject demographics

	MSC-NP group *N* = 27	Saline group *N* = 27
Female sex, n (%)	18 (67%)	20 (74%)
MS Subtype/EDSS block, n (%)		
SPMS/3.0–4.0	5 (19%)	6 (22%)
SPMS/4.5–5.5	5 (19%)	4 (15%)
SPMS/6.0	5 (19%)	5 (19%)
SPMS/6.5	5 (19%)	6 (22%)
PPMS/3.0–4.0	3 (11%)	3 (11%)
PPMS/4.5–5.5	1 (4%)	1 (4%)
PPMS/6.0	2 (7%)	1 (4%)
PPMS/6.5	1 (4%)	1 (4%)
Age at treatment start (years),		
Mean (SD) Median (IQR)	51 (7) 53 (44, 56)	49 (9) 49 (40, 57)
Disease duration (years),		
Mean (SD) Median (IQR)	12 (5) 12 (9, 17)	11 (6) 11 (6, 15)
Concomitant DMTs	RTX (*n* = 14) OCR (*n* = 8) NAT (*n* = 1) MMF/IVIG (*n* = 1) MTX (*n* = 2) none (*n* = 1)	RTX (*n* = 9) OCR (*n* = 7) NAT (*n* = 6) RTX/IVIG (*n* = 1) IVIG (*n* = 1) TR (*n* = 2) none (*n* = 1)

MSC-NP = mesenchymal stem cell-derived neural progenitors. MS = multiple sclerosis. SPMS = secondary progressive MS. PPMS = primary progressive MS. EDSS = Kurtzke expanded disability status scale. DMT = disease modifying therapy. RTX = rituximab. OCR = ocrelizumab. NAT = natalizumab. IVIG = intravenous immunoglobulin. TR = teriflunomide. MMF = mycophenolate mofetil. MTX = intrathecal methotrexate

Three subjects discontinued the study in year one (Fig. 1). One subject withdrew consent for personal reasons after one treatment, one subject discontinued after three treatments due to COVID-19 pandemic-related travel restrictions, and one subject exhibited previously undiagnosed hyperparathyroidism after six treatments and subsequently required parathyroidectomy, which was categorized as a serious adverse event unrelated to study intervention. All three discontinued subjects were coincidentally assigned to the MSC-NP group. An additional four subjects were discontinued in year two as detailed in Fig. 1. Due to missing outcome assessments in discontinued subjects, primary and secondary outcomes were analyzed in the per-protocol population, whereas safety outcomes were analyzed in the intent-to-treat population.

**Fig. 2:**
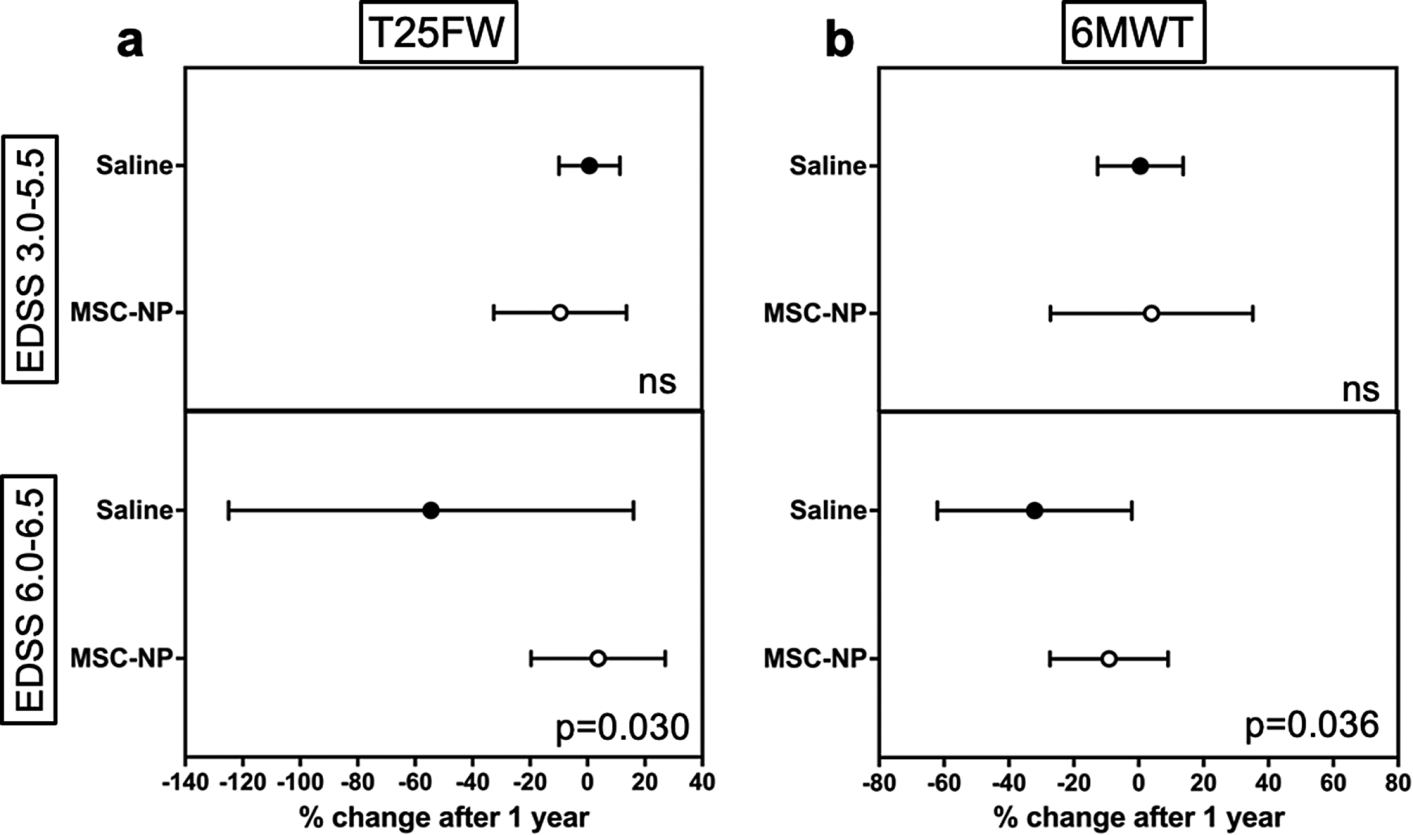
Walking outcomes after MSC-NP treatment compared to saline. Percentage change in (**a**) T25FW time and (**b**) 6MWT distance after one year of MSC-NP or saline treatments compared to baseline. Subjects were grouped by low EDSS (EDSS 3.0-5.5) (top graphs) or high EDSS (EDSS 6.0-6.5) (bottom graphs). Values represent mean and standard deviation. ns, not significant

The primary and secondary outcomes of the study are shown in Table 2. A significant difference in the pre-specified primary endpoint of EDSS Plus was not observed when comparing the MSC-NP-treated (33% improvement) to the saline-treated (37% improvement) group after one year (*p* = 0.666) (Table 2). Although the primary endpoint was not met, measures of ambulation function included as secondary outcomes supported efficacy of treatment. Post-hoc subgroup analysis showed that in subjects with higher baseline EDSS who require assistance for ambulation (EDSS 6.0-6.5), MSC-NP treatment was associated with a higher percentage change in both the T25FW test and the 6MWT compared to saline injection, (*p* = 0.030 and *p* = 0.036, respectively) (Table 2; Fig. 2). The difference reflected a worsening in walking speed and endurance in subjects with high EDSS treated with saline (Fig. 2). In the EDSS 6.0-6.5 subgroup, T25FW worsening, as defined by > 20% increase in walk time compared to baseline, occurred in the majority of subjects receiving saline (54%) compared to subjects receiving MSC-NPs (10%), however the difference did not reach significance (*p* = 0.074) (Table 2). No differences in other secondary outcomes such as 9HPT or MSFC were observed.

**Table 2:** Primary and secondary outcomes after year 1

		MSC-NP Group	Saline Group	*P* value
EDSS Plus improvement				
All subjects	n/N (%)	8/24 (33%)	10/27 (37%)	0.666
EDSS change				
All subjects	Mean (SD) Median (IQR)	-0.10 (0.53) 0.0 (-0.4, 0.0)	-0.26 (0.79) 0.0 (-0.5, 0.0)	0.483
T25FW improvement				
All subjects		4/24 (17%)	3/27 (11%)	0.693
Baseline EDSS 3.0-5.5 Baseline EDSS 6.0-6.5	n/N (%)	1/14 (7%) 3/10 (30%)	1/14 (7%) 2/13 (15%)	> 0.999 0.618
T25FW worsening				
All subjects		4/24 (17%)	7/27 (26%)	0.422
All subjects	n/N (%)	3/14 (21%) 1/10 (10%)	0/14 (0%) 7/13 (54%)	0.222 0.074
T25FW % change				
All subjects	Mean (SD) Median (IQR)	-3.9 (23.5) -5.2 (-12.6, 9.0)	-25.7 (56.1) -6.8 (-27.1, 4.8)	0.344
Baseline EDSS 3·0–5·5	Mean (SD) Median (IQR)	-9.3 (23.0) -5.2 (-11.8, 3.0)	0.9 (10.4) 1.5 (-6.5, 8.5)	0.306
Baseline EDSS 6·0–6·5	Mean (SD) Median (IQR)	3.7 (23.1) -5.2 (-11.9, 20.2)	-54.4 (70.5) -35.5 (-104.4, -8.8)	0.030
6MWT % change^a^				
All subjects	Mean (SD) Median (IQR)	-1.0 (27.3) -4.5 (-14.7, 8.2)	-15.0 (28.0) -10.7 (-24.1, 4.5)	0.222
Baseline EDSS 3·0–5·5	Mean (SD) Median (IQR)	4.3 (31.3) -7.3 (-12.3, 14.6)	0.8 (13.2) 0.5 (-11.0, 8.1)	0.804
Baseline EDSS 6·0–6·5	Mean (SD) Median (IQR)	-9.2 (18.2) -2.5 (-19.4, 0.0)	-32.1 (30.0) -26.9 (-41.5, -10.7)	0.036
9HPT % change, dominant hand				
All subjects	Mean (SD) Median (IQR)	-2.5 (19.1) 3.8 (-8.3, 6.7)	-4.6 (16.7) -3.1 (-11.6, 6.0)	0.470
9HPT % change, non-dominant hand				
All subjects	Mean (SD) Median (IQR)	-4.8 (27.1) -2.0 (-8.3, 9.5)	-4.5 (14.5) -1.8 (-8.0, 1.9)	0.693
MSFC, change in score from baseline				
All subjects	Mean (SD) Median (IQR)	-0.11 (0.24) 0.14 (0.05, 0.25)	-0.02 (0.35) -0.05 (-0.26, 0.21)	0.095
MSWS-12, change in score from baseline				
All subjects	Mean (SD) Median (IQR)	-0.9 (8.6) -1.7 (-5.0, 4.2)	2.2 (18.7) 0.0 (-10.0, 6.7)	0.619
PASAT, change in score from baseline				
All subjects	Mean (SD) Median (IQR)	4.0 (5.7) 4.0 (2.0, 8.0)	2.8 (9.1) 2.0 (-4.0, 6.0)	0.195

^a^Number of subjects in MSC-NP Year 1 group for 6MWT was *n* = 23. One subject in MSC-NP group could not perform walking tests (6MWT or T25FW) at the end of year 1 due to weakness related to infection (cholecystitis). Subject fully recovered at the time of 1.5 year assessment and T25FW time performed at 1.5 year was used in place of the missing 1 year assessment. 6MWT not performed at 1.5 year assessment

MSC-NP = mesenchymal stem cell-derived neural progenitors. EDSS = Kurtzke expanded disability status scale. T25FW = timed 25-foot walk. 6MWT = six-minute walk test. 9HPT = nine-hole peg test. MSFC = multiple sclerosis functional composite. MSWS-12 = twelve item MS walking scale. PASAT = paced auditory serial addition test

Bladder function was assessed by urodynamics testing before and after treatment. Subjects with normal bladder function at baseline and for the duration of the study were excluded from the analysis (*n* = 11). Of the subjects with neurogenic bladder dysfunction, we analyzed the PVR volume in 11 subjects in the placebo group before and after saline injections in year one, and 17 subjects before and after MSC-NP injections received in either year one or year two. The remaining subjects could not be included in the bladder function analysis due to noncompliance with urodynamics testing resulting in missing data. Noncompliance with urodynamics testing was due in large part to lack of access to the urology clinic during the COVID-19 pandemic.

**Fig. 3:**
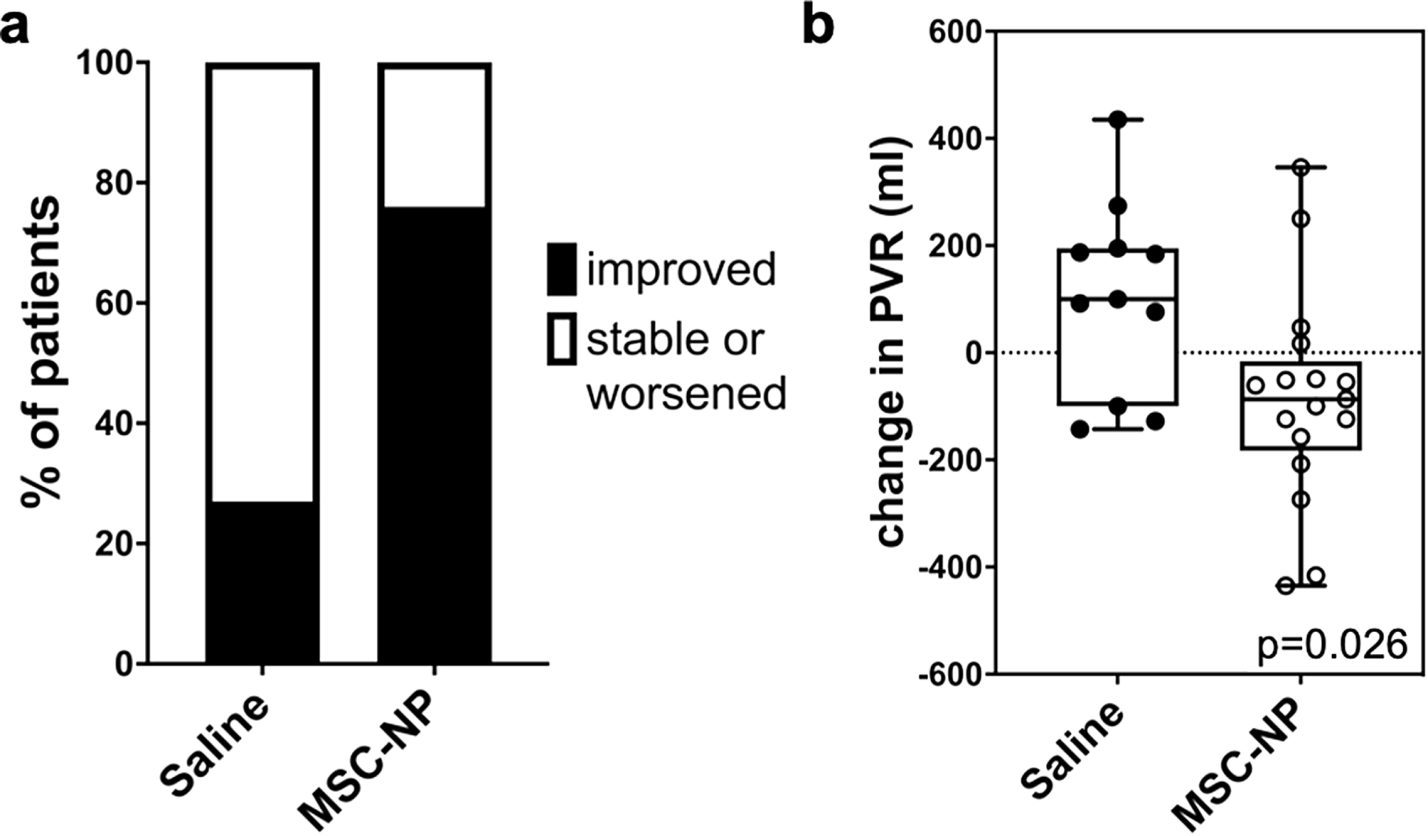
MSC-NP treatment was associated with improved bladder function and reduced PVR. (**a**) 76% (13/17) subjects treated with MSC-NPs demonstrated improved bladder function after one year, compared to 27% (3/11) subjects treated with saline. (**b**) PVR volume was measured before and after one year of either saline or MSC-NP injections. MSC-NP treatment was associated with a significant reduction in PVR volume (ml) compared to saline injections

We found that the majority (76%) of subjects analyzed demonstrated improved bladder function following MSC-NP treatment compared to 27% of subjects receiving saline (Fig. 3a). Despite both groups displaying similar average PVR volume at baseline (*p* = 0.12) (data not shown), the reduction in PVR volume was significantly greater after MSC-NP treatment compared to saline (*p* = 0.026) (Fig. 3b). These results are consistent with previous findings from our open-label phase I trial demonstrating improved bladder function in 50% of subjects receiving IT-MSC-NP treatment [13].

**Fig. 4:**
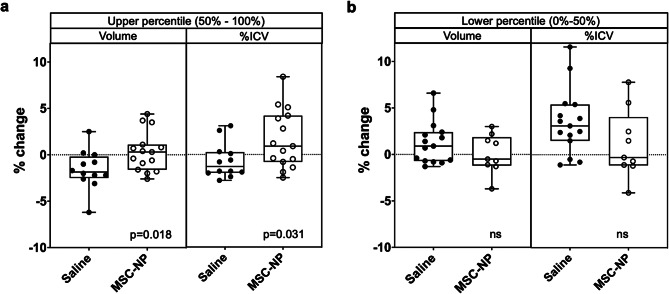
MSC-NP treatment was associated with preservation of grey matter atrophy. Subjects were grouped into either (**a**) the upper percentile (50–100%) or (**b**) the lower percentile (0–50%) based on their normative grey matter volume at baseline. In the upper percentile group, there was a significant difference in the percentage change of grey matter volume after one year of MSC-NP treatment compared to saline treatment when calculated as absolute grey matter volume or as a percentage of intra-cranial volume (%ICV). Percentage change was not different between MSC-NP and saline in the lower percentile group. ns, not significant

Individual MRI measures were analyzed as secondary outcomes in this study. No new T2 lesions or changes in total lesion disease burden were observed in brain, cervical or thoracic MRI scans (data not shown). As an exploratory outcome, we measured grey matter (GM) volume using Neuroquant imaging software, which compares individual volumes to a normative database taken as a percentage of total intracranial volume (%ICV) which is adjusted for age and gender. As a measure of brain atrophy, GM volume was compared between the MSC-NP and saline group. In a subgroup of subjects with normalized GM volume above the 50th percentile, saline treatment was associated with decreased GM volume after one year compared to MSC-NP treatment (*p* = 0.018) (Fig. 4 and Supplemental Fig. 1). The change in GM volume was not different between MSC-NP and saline treatment in the lower percentile group. These results suggest that in subjects with a lesser degree of brain atrophy at baseline, MSC-NP treatment was neuroprotective.

All adverse events (AEs) occurring during the entire two-year study period are shown in Table 3. Overall, there was a slightly higher number of AEs associated with MSC-NP injections. There were 76 AEs in 38 (70%) of 54 subjects receiving MSC-NP injections (39 AEs in 19 (70%) of 27 subjects in year one, and 37 AEs in 19 (70%) of 27 subjects in year two) compared to 42 AEs in 27 (53%) of 51 subjects during saline injections (20 AEs in 14 (52%) of 27 subjects in year one, and 22 AEs in 13 (54%) of 24 subjects in year two). A total of eight serious AEs occurred (Table 3), none of which were related to the study intervention. No hospitalizations were related to the study intervention and no deaths were reported.

**Table 3:** Adverse events

	MSC-NP Year 1 *N* = 27^a^	Saline Year 2 *N* = 24^b^	Saline Year 1 *N* = 27	MSC-NP Year 2 *N* = 27
Any AE, no. of patients (%)	19 (70%)	13 (54%)	14 (52%)	19 (70%)
Any Serious AE, no. of patients (%)	2 (7%)^c^	3 (13%)^d^	0 (0%)	3 (11%)^e^
Total no. of individual AEs	39	22	20	37
AEs by preferred term^f^, no. of patients (%)				
Urinary Tract Infection	4 (15%)	2 (8%)	3 (11%)	3 (11%)
Upper Respiratory Infection	1 (4%)	5 (21%)	2 (7%)	6 (22%)
Back pain	2 (7%)	0 (0%)	1 (4%)	1 (4%)
Generalized muscle weakness	2 (7%)	1 (4%)	4 (15%)	2 (7%)
Muscle weakness lower limb	2 (7%)	1 (4%)	1 (4%)	0 (0%)
Cerebrospinal fluid leakage	2 (7%)	0 (0%)	1 (4%)	1 (4%)
Graft vs. host disease^g^	0 (0%)	0 (0%)	0 (0%)	1 (4%)
Rash	0 (0%)	1 (4%)	0 (0%)	2 (7%)
Allergic reaction	0 (0%)	1 (4%)	0 (0%)	1 (4%)
Fever	1 (4%)	0 (0%)	0 (0%)	1 (4%)
Diarrhea	1 (4%)	0 (0%)	1 (4%)	0 (0%)
Neck pain	1 (4%)	0 (0%)	0 (0%)	1 (4%)
Neuralgia	2 (7%)	0 (0%)	1 (4%)	0 (0%)
Parasthesia	2 (7%)	0 (0%)	0 (0%)	0 (0%)
Skin infection	1 (4%)	0 (0%)	1 (4%)	0 (0%)
Spasticity	1 (4%)	1 (4%)	0 (0%)	0 (0%)
Trigeminal nerve disorder	1 (4%)	0 (0%)	0 (0%)	1 (4%)
Vertigo	1 (4%)	1 (4%)	0 (0%)	0 (0%)

^a^Total number of patients in the safety population (i.e. all subjects enrolled in the study who received any study injections, regardless of eventual removal from study)

^b^The 3 subjects who discontinued study in year 1 were not included in safety analysis in year 2

^c^(1) Severe urinary tract infection and urosepsis requiring hospitalization. Unrelated to the infection, subject was subsequently removed from study due to COVID-19-related travel restrictions and study noncompliance on behalf of the subject. (2) Previously undiagnosed hyperparathyroidism requiring parathyroidectomy. Chronic hypercalcemia contributed to urinary tract infection and sepsis. Subject was removed from study

^d^(1) Pulmonary embolism and deep vein thrombosis associated with long COVID. Subject was removed from study. (2) COVID-19 infection and severe urinary tract infection. (3) Cholecystitis requiring cholecystectomy

^e^(1) Lung infection associated with long COVID. (2) Acute appendicitis complicated by bowel perforation. (3) Bilateral muscle weakness associated with MS progression

^f^Only AEs occurring in 2 or more subjects listed

^g^Occurred in 1 subject immediately following each MSC-NP injection

MSC-NP = mesenchymal stem cell-derived neural progenitors. AE = adverse event

Similar to the safety profile reported in our phase I trial, we observed an increase in the frequency of headaches in the two-day period following MSC-NP injections (Supplemental Table 1) [13]. The headache frequency in the MSC-NP treated group (year one and year two) was 34% in the two-day period following MSC-NP injections compared to 15% frequency in the two-day period following saline injections. The frequency of headaches occurring one week or one month after each procedure was the same (18%) in both groups. The overall headache frequency is significantly lower than that observed in the phase I trial, likely due to the use of prophylactic acetaminophen in the phase II protocol. Other than headache, only one AE was considered related to the MSC-NP injection, where one subject experienced a graft-vs-host-related rash immediately following each MSC-NP injection (Table 3). For each graft-vs-host event, the subject recovered without treatment.

**Fig. 5:**
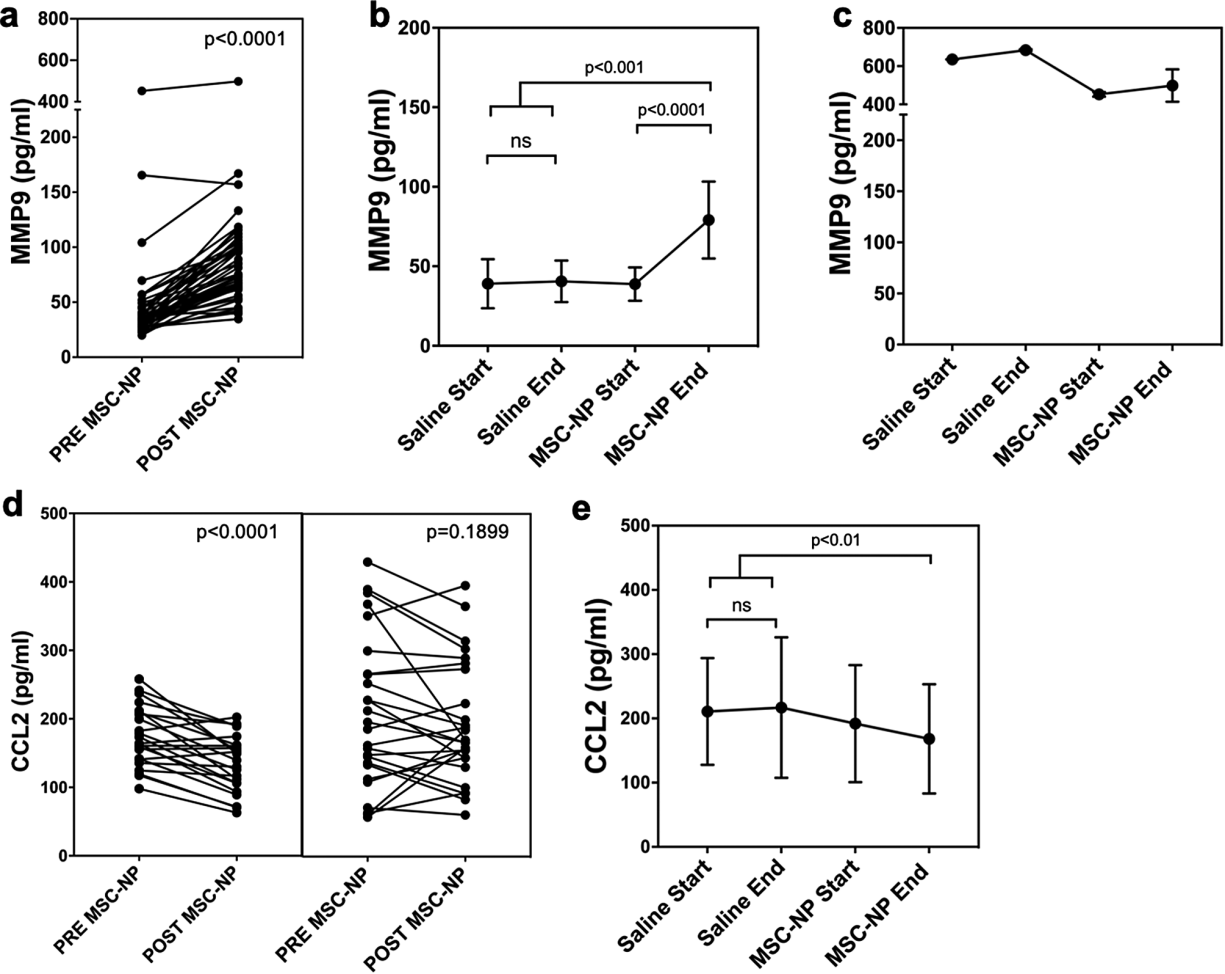
Increased MMP9 and decreased CCL2 in CSF following MSC-NP treatment. (**a**) CSF MMP9 levels pre- and post-MSC-NP treatment in all 50 phase II subjects. (**b**) Longitudinal changes in CSF MMP9 levels in the 26 subjects who received saline in year one and MSC-NPs in year two. One outlier subject exhibited much higher CSF MMP9 levels and is graphed separately in (**c**). MMP9 levels were unchanged over year one (saline) and significantly increased after year two following MSC-NP treatment. Outlier was included in the statistical analysis. (**d**) CSF CCL2 levels pre- and post-MSC-NP treatment in all trial subjects divided into responder (*n* = 23) (left panel) and non-responder (*n* = 26) (right panel) subgroups. Biomarker values from one subject was removed from analysis due to missing outcome data. Decreased CCL2 was statistically significant in responders (left panel) but not in non-responders (right panel). (**e**) Longitudinal changes in CSF CCL2 levels in the 26 subjects who received placebo in year 1 and MSC-NPs in year 2. CCL2 levels were unchanged over year one (placebo) and significantly decreased in year two following MSC-NP treatment

CSF biomarker analysis identified 146 proteins found to be significantly increased or decreased following MSC-NP treatment (Supplemental Table 2), and two biomarkers, MMP9 and CCL2, were independently validated. We found that MMP9 was significantly increased following MSC-NP treatment in all subjects (Fig. 5a). Longitudinal analysis demonstrated that MMP9 levels were stable during saline injections and were significantly elevated following MSC-NP treatment (Fig. 5b). Conversely, CCL2 levels were significantly decreased in CSF following MSC-NP treatment, specifically in the subgroup of patients demonstrating a clinical response after MSC-NP injection (23 of 49 subjects, or 47%) (Fig. 5d). CCL2 levels were stable during saline injections and were significantly decreased following MSC-NP treatment in year 2 (Fig. 5e). The increase of MMP9 and decrease of CCL2 in CSF following MSC-NP treatment provides a biochemical indicator of MSC-NP treatment, possibly reflecting treatment efficacy in patients with MS.

## Discussion

The current study adds to a body of evidence demonstrating safety and tolerability of intrathecally administered autologous MSC or MSC-derived products such as MSC-NPs in patients with MS [13–15, 18–20]. The study population consisted of ambulatory, non-relapsing SPMS and PPMS patients who did not have MRI evidence of active disease. The study did not meet the primary efficacy endpoint of a positive change in EDSS Plus when comparing MSC-NP and saline groups due to a large unanticipated placebo effect. Recent analysis of the placebo arms of the ASCEND and IMPACT trials in SPMS revealed a high rate of EDSS improvement over time (22% and 14% improvement after 1 year, respectively, compared to the T25FW outcome which remained stable below 10% [21]. The high placebo-associated improvement rate in EDSS may be due to the interval-scaled nature of EDSS assessment and low inter-rater and intra-rater reliability of this outcome, specifically in progressive MS [21, 22]. The unreliability of EDSS as an outcome measure in trials involving progressive MS patients should also be taken into consideration when concluding the degree of efficacy of MSC-NP treatment during our phase I trial, which showed 40% improvement in EDSS [13]. Aside from EDSS, both the phase I trial and the current phase II trial demonstrated improvements in objective and clinically relevant outcome measures, namely T25FW and bladder function, supporting the clinical potential of this therapeutic approach [13].

In the current study, analysis of secondary outcome measures demonstrated significant efficacy of MSC-NP treatment compared to the control. Differences in walking outcomes were specifically observed in patients needing assistive devices for ambulation (EDSS of 6.0-6.5) in both the short-timed speed test and the longer duration endurance test, both of which showed significant benefit of MSC-NP treatment compared to placebo. Despite the statistical limitations of a subgroup analysis that was not pre-specified, the treatment efficacy observed in the EDSS 6.0-6.5 group is clinically meaningful given that ambulation is most affected in patients using assistive devices.

Two previous clinical studies have investigated the efficacy of IT injections of MSCs or MSC-derived products in patients with MS. In one study, a single IT dose of autologous MSCs was tested compared to IV MSC and sham injection [23]. Despite some clinical benefit associated with MSC injection, the unusual crossover design and the inclusion of actively progressing patients rendered the outcomes difficult to interpret [23]. In the other study, multiple IT injections of neurotrophic factor secreting MSCs in relapse-free progressive patients was associated with improved clinical outcomes [24]. Although the trial lacked a true placebo control, they found that 19% of subjects demonstrated improved walking in the T25FW following treatment which suggested improvement compared to < 5% matched subjects from an independent control cohort [24]. Walking improvement was comparable to our study, where we found that 17% of subjects demonstrated improvement in T25FW overall, and 30% of subjects improved in the EDSS 6.0-6.5 group.

Efficacy of MSC-NP treatment was also seen in relation to bladder dysfunction. A subset of our patients exhibited disabling bladder dysfunction at study entry as established by history and verified by urodynamic testing. In these patients, significant improvement was seen in the treatment group in more than 75% of patients with improved bladder emptying as determined by a > 20% decrease in PVR. The objective improvement in urodynamics measurement was associated with improved urinary urgency, decreased double voiding, and decreased frequency of nocturia, reflecting a clinically meaningful improvement in bladder symptoms. Despite the limitations caused by missing data related to pandemic disruptions, these results extended and supported similar outcomes from our previous phase I study [13]. The observed bladder function improvement in addition to a positive benefit in ambulatory measures is noteworthy because it depicts possible benefit in two distinct spinal cord pathways.

GM atrophy based on MRI is a correlate of the neurodegenerative process that contributes to disease progression in MS [25, 26]. The association with cognition dysfunction and fatigue underscores the clinical relevance of cortical GM volume measurements compared to whole brain or white matter volume [26–29]. In addition, multiple studies have demonstrated a correlation between GM atrophy with disease progression and disease severity [25, 28, 30, 31]. Furthermore, reduced rate of GM atrophy has been explored as an outcome for neuroprotective treatments in MS. For example, the therapeutic effect of ibudilast was shown to significantly impact GM atrophy in progressive MS [32]. We found that MSC-NP treatment correlated with reduced rate of cortical GM volume loss in a subgroup of patients with less impacted brain atrophy at baseline. We did not observe a correlation between percentage of GM volume change and change in clinical cognitive or disability score, suggesting a subclinical impact of MSC-NP treatment on the neurodegenerative process in the gray matter. The interpretation of these findings are limited by the low number of subjects in the subgroup analysis. Interestingly, a recent open-label trial testing the safety of brain-derived neural stem cells in patients with advanced MS also found a dose-dependent correlation with GM volume change, suggesting similar neuroprotective effects of intrathecal cell transplantation in MS [33].

Unbiased proteomic screening of CSF revealed significant changes in the level of MMP9 and CCL2 following MSC-NP treatment. Notably, we did not observe any changes in CSF neurofilament light (NfL) levels, in contrast to a previous study which showed lower NfL levels following IT MSC injections [34]. MMP9 is a matrix metalloproteinase that plays a role in developmental and pathological processes through its functions in extracellular matrix remodeling and cleavage of various substrates. In RRMS, MMP9 activity at the blood brain barrier plays a role in modulating the neuroinflammatory response by facilitating leukocyte migration and modulating local cytokine/chemokine response [35]. As such, MMP9 levels have been shown to be elevated in CSF in RRMS patients with MRI evidence of disease activity [36, 37]. In contrast, MMP9 also plays a beneficial role in repair and regeneration of the CNS by promoting synaptic remodeling, neurogenesis, and remyelination [38]. Specifically, MMP9 has been shown to facilitate oligodendrocyte maturation during remyelination following lysolecithin-induced demyelinating injury through cleavage of inhibitory proteoglycans [39]. The observed increase in MMP9 was specific to MSC-NP transplantation regardless of clinical response, suggesting that MSC-NPs may directly target cells producing MMP9, which may include astrocytes, microglia/macrophages, and endothelial cells [38]. Further research using in vitro and in vivo models of MS will be required to investigate the mechanisms by which MMP9 is upregulated in response to MSC-NP treatment. Given the small sample size of the current study, validation of these biomarkers in a larger cohort of patients will be required. Interestingly, elevated CSF MMP9 was also observed following transplantation of fetal neural stem cells in patients with MS [33], further implicating MMP9 as a biomarker of the regenerative response associated with intrathecal cell therapy.

MSC-NP treatment is also associated with reduced CSF levels of the chemokine CCL2. CCL2 is a pro-inflammatory chemoattractant that regulates neuroinflammation and is implicated in neuroinflammatory diseases including MS [40]. In MS brain autopsy tissue, CCL2 expression colocalizes with microglia/macrophages and with hypertrophic astrocytes in demyelinated lesions, identifying two possible CNS sources of CCL2 that may be responding to MSC-NP injections [41, 42]. In a recent study, MSC-NPs suppressed microglial activation and reduce CCL2 levels in vitro, further supporting the hypothesis that microglia may be a therapeutic target of MSC-NP treatment [11]. CSF biomarker analysis of our phase I trial demonstrated a similar decrease in CSF levels of CCL2 following IT MSC-NP injections [14]. The finding that decreased CCL2 is more pronounced in treatment responders indicates that it may serve as a useful surrogate biomarker of therapeutic response and supports the measurement of CCL2 in future trials to identify treatment responders within a shorter time period. We hypothesize that the reduced CCL2 in response to MSC-NP treatment indicates that microglia may play a more prominent role in disease progression in a subpopulation of MS patients. Future studies are required to elucidate the mechanisms underlying the microglial response to MSC-NP treatment, including the use of novel neuroimaging approaches to detect microglial activity in MS patients treated with MSC-NPs [43].

## Conclusions

The study is the first randomized double-blind phase II clinical trial investigating intrathecal MSC-NP cell therapy compared to a placebo control in a population of MS patients with progressive disease. The results demonstrate that when compared to saline, IT MSC-NP treatment was associated with improved walking ability in the subset of patients requiring an aid for walking. Although the primary outcome of EDSS-based improvement was not met, the significant improvement in secondary walking outcomes addresses an unmet need in MS patients with progressive disability. Furthermore, IT MSC-NP injection was associated with improved bladder function which is a relevant quality of life issue in people with MS. In addition, we found indirect evidence of a neuroprotective effect as seen by brain MRI cortical gray matter volume changes. Furthermore, our study also adds to existing evidence demonstrating the safety and tolerability of multiple intrathecal injections of cell doses. The clinical observations were associated with significant biological changes in CSF biomarkers following treatment. Our study provides multiple lines of clinical and laboratory evidence that demonstrate efficacy of IT MSC-NP therapy in progressive MS. Future studies employing ambulatory measures as primary endpoints and investigating optimal dosing of MSC-NPs are needed.

### Electronic supplementary material

Below is the link to the electronic supplementary material.

Supplementary Material 1

Supplementary Material 2

Supplementary Material 3

Supplementary Material 4

## Data Availability

De-identified sections of the dataset will be available from the corresponding author upon reasonable request from the time of publication. On request, additional documents including study protocol, statistical analysis plan, and informed consent will be made available.

## Abbreviations

MSC: Mesenchymal stem cell
MSC-NP: Mesenchymal stem cell-derived neural progenitor
MS: Multiple sclerosis
RRMS: Relapsing-remitting multiple sclerosis
SPMS: Secondary progressive multiple sclerosis
PPMS: Primary progressive multiple sclerosis
IT: Intrathecal
CSF: Cerebrospinal fluid
EDSS: Kurtzke expanded disability status scale
DMT: Disease modifying therapy
T25FW: Timed 25-foot walk
6MWT: Six-minute walk test
9HPT: Nine-hole peg test
MSFC: Multiple sclerosis functional composite
MSWS-12: Twelve item MS walking scale
PASAT: Paced auditory serial addition test
MRI: Magnetic resonance imaging
MMP9: Matrix metalloproteinase 9
CCL2: C-C motif chemokine ligand 2
PVR: Post-void residual
AE: Adverse event
GM: Grey matter
ICV: Intracranial volume
