# Supplementary material for: Efficacy of intrathecal mesenchymal stem cell-neural progenitor therapy in progressive MS: results from a phase II, randomized, placebo-controlled clinical trial
Source: Stem Cell Res Ther. 2024 May 23;15:151. doi: 10.1186/s13287-024-03765-6 (PMC11119709; doi:10.1186/s13287-024-03765-6)
Supplement: Supplementary file 1 — Supplementary Material 1 [file 13287_2024_3765_MOESM1_ESM.docx]

**Supplemental Table 1. Headache Frequency**

|  | MSC-NP Year 1 | Saline Year 2 | Saline Year 1 | MSC-NP Year 2 |
| --- | --- | --- | --- | --- |
| Total # of treatments^a^, N | 155 | 138 | 162 | 162 |
| Headache occurrence after each treatment  n (% of treatments)  2 days  7 days  30 days | 58 (37%)  11 (7%)  12 (8%) | 27 (20%)  10 (7%)  17 (12%) | 19 (12%)  15 (9%)  13 (8%) | 51 (31%)  18 (11%)  15 (9%) |
| Total headaches, N | 81 | 54 | 47 | 84 |
| Headache Severity, n (% of headaches) |  |  |  |  |
| Mild/Moderate | 74 (91%) | 52 (96%) | 46 (98%) | 69 (82%) |
| Severe | 7 (9%) | 2 (4%) | 1 (2%) | 15 (18%) |

^a^Total number of treatments (placebo or MSC-NP injection) in each year in the safety population (i.e. all subjects enrolled in the study who received any study injections, regardless of eventual removal from study).
