# Supplementary material for: Efficacy of intrathecal mesenchymal stem cell-neural progenitor therapy in progressive MS: results from a phase II, randomized, placebo-controlled clinical trial
Source: Stem Cell Res Ther. 2024 May 23;15:151. doi: 10.1186/s13287-024-03765-6 (PMC11119709; doi:10.1186/s13287-024-03765-6)
Supplement: Supplementary file 2 — Supplementary Material 2 [file 13287_2024_3765_MOESM2_ESM.docx]

**Supplemental Methods**

**Efficacy of Intrathecal Mesenchymal Stem Cell-Neural Progenitor Therapy in Progressive MS: Results from a Phase II, Randomized, Placebo-Controlled Clinical Trial**

**Harris, V.K. et al**

**Preparation of autologous MSC-NP cells**

# Cell manufacturing was performed in a cGMP facility at the Tisch MS Research Center of New York. To generate MSC-NPs, the mononucleated cell fraction from a 20 ml sternal bone marrow aspirate was isolated using Ficoll gradient density centrifugation, and mononucleated cells were plated in MSC growth medium (Lonza, MD, USA) supplemented with 5% Plasate™ human platelet lysate (New York Blood Center), 2 U/ml heparin and 2 mM GlutaMAX™I CTS™ (Life Technologies, NY, USA). Cells were incubated in a humidified 37^o^C incubator at 5% CO_2_ and 5% O_2_ inside an ISO-5 closed aseptic isolator system (Xvivo System® model X2, Biospherix, Ltd.). MSCs were isolated based on plastic adherence, passaged when they reached 80% confluency using CTS™ TrypLE™ Select Enzyme (ThermoFisher Scientific) and replated at a density of 2000-3000 cells/cm^2^. At passage 2 and passage 3, MSCs were cryopreserved in PRIME-XV FreezIS DMSO-Free freezing media (IrvineScientific), generating a stock of cells sufficient for multiple subsequent expansions.

# Each batch of bone marrow MSCs was tested and released according to the following criteria: (1) Confirmation of MSC attachment and spindle-shaped morphology, (2) Ex vivo expansion and cryopreservation at passage 2 and 3, (3) Sterility testing of passage 1 and passage 2 cells by direct inoculation method according to USP <71> sterility test, (4) Normal karyotype with no clonal abnormalities as determine by G-banded chromosome analysis (performed by Creative Bioarray), (5) Confirmation of cell surface marker expression using MSC Phenotyping Cocktail Kit, anti-human, REAfinity™ (Miltenyi Biotech), (6) Confirmation of adipogenic differentiation in Adipogenic Differentiation BulletKit^TM^Medium (Lonza) followed by Oil-Red O staining, and (7) confirmation of osteogenic differentiation in Osteogenic Differentiation BulletKit^TM^Medium (Lonza) followed by Alizarin Red staining.

MSC-NPs were generated from MSCs prior to each treatment. A portion of cryopreserved MSCs were thawed and expanded for two more passages as described above. To generate MSC-NPs, expanded MSCs were plated in low-adherence flasks in neural progenitor maintenance medium (Lonza) supplemented with 20 ng/ml each of epidermal growth factor (EGF) and basic fibroblast growth factor (bFGF). Media was changed every 2-3 days for 2 weeks. Floating MSC-NP cell clusters were visible after 2-5 days. Just prior to the injection, MSC-NPs were collected and briefly resuspended in CTS™ TrypLE™ Select Enzyme (ThermoFisher Scientific) to obtain a single cell suspension. Cells were washed twice in preservative-free saline, and cell number and viability were determined by AO/PI staining using an automated cell counter (Nexelcom Biosciences, LLC).

Each batch of MSC-NPs was released according to the following final product release specifications: (1) Adequate expansion (specification >20 x 10^6^ cells, average >100 x 10^6^ cells) of MSCs from cryopreserved stocks, (2) Sterility testing of the last media change (≥48 hours prior to final product release) by direct inoculation method according to USP <71> sterility test, (3) Sterility testing of final product by gram stain, (4) Confirmation of “neurosphere” morphology and identity, (4) Final product viability >70% by AO/PI staining, and (5) cell number up to 10 x 10^6^ cells per dose. Post-release testing included (1) Sterility of in-process sample (inoculated ≥48 hours prior to final product release) after 14 days according to USP <71> sterility test, (2) Sterility of final product sample after 14 days according to USP <71> sterility test, (3) Purity of final product by endotoxin testing, and (4) Potency testing by gene expression analysis of final product MSC-NPs compared to MSCs from which they were derived. Gene expression testing was performed using TaqMan® Gene Expression Assays (Thermo Fisher Scientific) and specified >2-fold down-regulation of mesodermal lineage markers smooth muscle isoform of alpha 2 actin (ACTA2) (Assay ID: Hs00426835_g1) and CD90 (THY1) (Assay ID: Hs00264235_s1) and >2-fold up-regulation of C-X-C motif chemokine receptor 4 (CXCR4) (Assay ID: Hs00607978_s1), hepatocyte growth factor (HGF) (Assay ID: Hs00300159_m1), leukemia inhibitory factor (LIF) (Assay ID: Hs00171455_m1), nestin (NES) (Assay ID: Hs00707120_s1), Sry-box 2 (SOX2) (Assay ID: Hs00602736_s1), and toll like receptor 2 (TLR2) (Assay ID: Hs01872448_s1). Gene expression was normalized to IPO8 endogenous control (Hs00183533_m1) and relative quantification was determined by delta delta Ct analysis of MSC-NPs compared to the MSCs using RQ Manager software (Applied Biosystems).

Bone marrow MSC batches were generated for all 54 subjects in the trial. For one subject, the first batch of MSCs failed due to equipment malfunction, however a repeat of bone marrow aspiration and MSC expansion was successful. For another subject, the first batch of MSCs passed released testing but failed to expand in sufficient numbers of cells to generate a sufficient number of MSC-NPs. Repeat bone marrow aspiration yielded the same outcome, and thus the subject was removed from study. A total of 318 individual batches of MSC-NPs were generated during the trial. Of these, 7 batches (2.2%) were rejected due to contamination detected in-process. Viability of the majority (93%) of MSC-NP batches at the time of injection was ≥90% and in a minority of batches (7%) viability was between 80%-89%. At each passage, the population doubling time (PDT) of MSCs was calculated as PDT = (t*log2)/(logN_2_-logN_1_) where t = number of days in culture, N_1_ = number of cells seeded and N_2_ = number of cells harvested. The average PDT of expanded MSCs during passage 2 and passage 3 was 2.3 days (range 1.7 to 3.6) and 2.6 days (range 1.4 to 5.7), respectively. Four MSC lines were expanded up to passage 4, where the average population doubling time was 2.8 days. We did not observe any correlation between PDT and subject age, disease subtype, or disease duration.
