# Supplementary material for: Efficacy of intrathecal mesenchymal stem cell-neural progenitor therapy in progressive MS: results from a phase II, randomized, placebo-controlled clinical trial
Source: Stem Cell Res Ther. 2024 May 23;15:151. doi: 10.1186/s13287-024-03765-6 (PMC11119709; doi:10.1186/s13287-024-03765-6)
Supplement: Supplementary file 3 — Supplementary Material 3 [file 13287_2024_3765_MOESM3_ESM.docx]

**Supplemental Table 2. List of 146 CSF proteins found to be differentially regulated post-MSC-NP treatment compared to baseline. Proteins listed in order of log2 fold regulation.**

| **Target** | **Entrez Gene Symbol** | **Target Full Name** | **Log2_FC** | **pval** | **FDR** | **PRE_lsmean** | **POST_lsmean** |
| --- | --- | --- | --- | --- | --- | --- | --- |
| **MMP-9** | **MMP9** | Matrix metalloproteinase-9 | 1.2547 | 7.63E-17 | 5.56E-13 | 9.47 [9.26-9.68] | 10.73 [10.51-10.94] |
| **GPDA** | **GPD1** | Glycerol-3-phosphate dehydrogenase [NAD(+)], cytoplasmic | 0.5283 | 1.48E-03 | 2.86E-01 | 7.67 [7.40-7.94] | 8.20 [7.92-8.47] |
| **Chitotriosidase-1** | **CHIT1** | Chitotriosidase-1 | 0.3138 | 5.64E-07 | 1.37E-03 | 10.08 [9.62-10.54] | 10.39 [9.93-10.86] |
| **CK-MB** | **CKB\|CKM** | Creatine kinase M-type:Creatine kinase B-type heterodimer | 0.3098 | 4.74E-03 | 4.37E-01 | 9.75 [9.50-10.01] | 10.06 [9.80-10.33] |
| **F16P1** | **FBP1** | Fructose-1,6-bisphosphatase 1 | 0.2820 | 4.54E-04 | 2.07E-01 | 10.46 [10.25-10.67] | 10.74 [10.53-10.96] |
| **MDC** | **CCL22** | C-C motif chemokine 22 | 0.2450 | 6.83E-03 | 4.85E-01 | 7.13 [6.93-7.33] | 7.37 [7.17-7.58] |
| **MMP-12** | **MMP12** | Macrophage metalloelastase | 0.1769 | 3.59E-09 | 1.31E-05 | 6.91 [6.78-7.04] | 7.09 [6.96-7.22] |
| **CK-MM** | **CKM** | Creatine kinase M-type | 0.1706 | 6.26E-03 | 4.85E-01 | 8.47 [8.34-8.60] | 8.64 [8.51-8.77] |
| **ACO13** | **ACOT13** | Acyl-coenzyme A thioesterase 13 | 0.1241 | 8.59E-03 | 4.85E-01 | 6.79 [6.68-6.89] | 6.91 [6.80-7.02] |
| **Tissue transglutaminase** | **TGM2** | Protein-glutamine gamma-glutamyltransferase 2 | 0.0928 | 4.24E-04 | 2.07E-01 | 8.00 [7.92-8.09] | 8.10 [8.01-8.18] |
| **MMP-8** | **MMP8** | Neutrophil collagenase | 0.0761 | 1.18E-03 | 2.69E-01 | 7.12 [7.05-7.20] | 7.20 [7.13-7.27] |
| **NPM1** | **NPM1** | Nucleophosmin | 0.0417 | 1.42E-03 | 2.86E-01 | 8.05 [8.03-8.08] | 8.10 [8.07-8.12] |
| **LYN** | **LYN** | Tyrosine-protein kinase Lyn | 0.0398 | 9.37E-03 | 4.85E-01 | 7.88 [7.84-7.93] | 7.92 [7.88-7.97] |
| **LIMD2** | **LIMD2** | LIM domain-containing protein 2 | 0.0398 | 4.16E-03 | 4.24E-01 | 7.98 [7.95-8.01] | 8.02 [7.99-8.05] |
| **NADPH-P450 Oxidoreductase** | **POR** | NADPH--cytochrome P450 reductase | 0.0389 | 9.46E-03 | 4.85E-01 | 8.50 [8.47-8.54] | 8.54 [8.51-8.58] |
| **STAR5** | **STARD5** | StAR-related lipid transfer protein 5 | 0.0357 | 3.67E-03 | 4.09E-01 | 7.91 [7.88-7.94] | 7.94 [7.91-7.97] |
| **BARK1** | **GRK2** | beta-adrenergic receptor kinase 1 | 0.0354 | 2.32E-03 | 3.52E-01 | 7.65 [7.63-7.68] | 7.69 [7.66-7.72] |
| **LIGHT** | **TNFSF14** | Tumor necrosis factor ligand superfamily member 14 | 0.0342 | 5.81E-03 | 4.85E-01 | 7.69 [7.64-7.74] | 7.72 [7.67-7.78] |
| **Activin AC** | **INHBA\| INHBC** | Inhibin beta A chain:Inhibin beta C chain heterodimer | 0.0335 | 8.45E-03 | 4.85E-01 | 6.54 [6.51-6.57] | 6.57 [6.54-6.60] |
| **SH21A** | **SH2D1A** | SH2 domain-containing protein 1A | 0.0332 | 5.65E-03 | 4.79E-01 | 8.07 [8.04-8.09] | 8.10 [8.08-8.12] |
| **FA92A** | **FAM92A** | Protein FAM92A | 0.0326 | 9.03E-03 | 4.85E-01 | 7.22 [7.20-7.24] | 7.25 [7.24-7.27] |
| **ARSB** | **ARSB** | Arylsulfatase B | 0.0313 | 5.07E-03 | 4.42E-01 | 8.15 [8.11-8.18] | 8.18 [8.14-8.21] |
| **ARMC5:ARM 2** | **ARMC5** | Armadillo repeat-containing protein 5:Armadillo Repeat Domain 2 | 0.0257 | 4.02E-03 | 4.19E-01 | 8.00 [7.97-8.04] | 8.03 [7.99-8.06] |
| **PDRG1** | **PDRG1** | p53 and DNA damage-regulated protein 1 | 0.0253 | 7.50E-03 | 4.85E-01 | 7.17 [7.15-7.19] | 7.20 [7.18-7.21] |
| **CATE** | **CTSE** | Cathepsin E | 0.0251 | 9.15E-03 | 4.85E-01 | 8.05 [8.03-8.07] | 8.07 [8.05-8.09] |
| **GBA3** | **GBA3** | Cytosolic beta-glucosidase | 0.0216 | 5.49E-03 | 4.71E-01 | 7.86 [7.85-7.87] | 7.88 [7.87-7.90] |
| **GGE2D** | **GAGE2D** | G antigen 2D | -0.0172 | 8.88E-03 | 4.85E-01 | 7.82 [7.81-7.83] | 7.80 [7.79-7.81] |
| **ATP23** | **ATP23** | Mitochondrial inner membrane protease ATP23 homolog | -0.0223 | 1.24E-04 | 1.13E-01 | 8.36 [8.35-8.38] | 8.34 [8.33-8.36] |
| **FCRLA** | **FCRLA** | Fc receptor-like A | -0.0274 | 2.86E-03 | 3.66E-01 | 6.67 [6.65-6.68] | 6.64 [6.63-6.66] |
| **TAF12** | **TAF12** | Transcription initiation factor TFIID subunit 12 | -0.0279 | 2.71E-03 | 3.53E-01 | 7.02 [7.00-7.03] | 6.99 [6.98-7.00] |
| **TACT** | **CD96** | T-cell surface protein tactile | -0.0308 | 3.51E-03 | 4.09E-01 | 8.10 [8.08-8.11] | 8.07 [8.05-8.08] |
| **UGT 1A1** | **UGT1A1** | UDP-glucuronosyltransferase 1-1 | -0.0343 | 4.19E-03 | 4.24E-01 | 7.01 [6.98-7.03] | 6.97 [6.95-7.00] |
| **T-plastin** | **PLS3** | Plastin-3 | -0.0351 | 7.70E-03 | 4.85E-01 | 7.30 [7.27-7.32] | 7.26 [7.24-7.29] |
| **Calbindin D28** | **CALB1** | Calbindin | -0.0359 | 2.01E-03 | 3.38E-01 | 7.04 [7.02-7.06] | 7.00 [6.98-7.03] |
| **MAEA** | **MAEA** | Macrophage erythroblast attacher | -0.0366 | 7.23E-03 | 4.85E-01 | 6.76 [6.71-6.81] | 6.72 [6.67-6.77] |
| **ADAT2** | **ADAT2** | tRNA-specific adenosine deaminase 2 | -0.0379 | 7.63E-03 | 4.85E-01 | 7.00 [6.97-7.04] | 6.96 [6.93-7.00] |
| **MAGD1** | **MAGED1** | Melanoma-associated antigen D1 | -0.0387 | 9.26E-03 | 4.85E-01 | 5.98 [5.94-6.01] | 5.94 [5.90-5.97] |
| **ANRA2** | **ANKRA2** | Ankyrin repeat family A protein 2 | -0.0425 | 4.46E-03 | 4.24E-01 | 7.11 [7.08-7.14] | 7.07 [7.04-7.10] |
| **TPC6B** | **TRAPPC6B** | Trafficking protein particle complex subunit 6B | -0.0426 | 6.45E-03 | 4.85E-01 | 7.91 [7.88-7.93] | 7.86 [7.84-7.89] |
| **MO4L2** | **MORF4L2** | Mortality factor 4-like protein 2 | -0.0429 | 5.95E-03 | 4.85E-01 | 8.60 [8.56-8.65] | 8.56 [8.52-8.61] |
| **CDK20** | **CDK20** | Cyclin-dependent kinase 20 | -0.0437 | 6.54E-03 | 4.85E-01 | 8.15 [8.12-8.17] | 8.10 [8.07-8.13] |
| **RTN1** | **RTN1** | Reticulon-1 | -0.0459 | 2.04E-03 | 3.38E-01 | 8.24 [8.21-8.26] | 8.19 [8.17-8.22] |
| **TPRKB** | **TPRKB** | EKC/KEOPS complex subunit TPRKB | -0.0464 | 2.56E-03 | 3.52E-01 | 7.27 [7.24-7.30] | 7.23 [7.19-7.26] |
| **CLCA2** | **CLCA2** | Calcium-activated chloride channel regulator 2 | -0.0471 | 8.01E-03 | 4.85E-01 | 7.92 [7.87-7.97] | 7.87 [7.83-7.92] |
| **AKIR2** | **AKIRIN2** | Akirin-2 | -0.0485 | 8.27E-03 | 4.85E-01 | 7.48 [7.41-7.56] | 7.44 [7.36-7.51] |
| **PCDA4** | **PCDHA4** | Protocadherin alpha-4 | -0.0491 | 7.99E-03 | 4.85E-01 | 6.94 [6.84-7.05] | 6.89 [6.79-7.00] |
| **CD79A** | **CD79A** | B-cell antigen receptor complex-associated protein alpha chain | -0.0495 | 4.49E-03 | 4.24E-01 | 6.38 [6.35-6.42] | 6.33 [6.30-6.37] |
| **MAGE-10** | **MAGEA10** | Melanoma-associated antigen 10 | -0.0496 | 4.88E-03 | 4.39E-01 | 7.64 [7.58-7.71] | 7.59 [7.53-7.66] |
| **DDIT3** | **DDIT3** | DNA damage-inducible transcript 3 protein | -0.0529 | 7.83E-03 | 4.85E-01 | 6.35 [6.32-6.38] | 6.30 [6.27-6.33] |
| **SPT46** | **SPATA46** | Spermatogenesis-associated protein 46 | -0.0535 | 9.87E-03 | 4.93E-01 | 7.98 [7.92-8.03] | 7.92 [7.87-7.98] |
| **ERMIN** | **ERMN** | Ermin | -0.0541 | 8.43E-03 | 4.85E-01 | 7.62 [7.53-7.71] | 7.57 [7.47-7.66] |
| **TMM8B** | **TMEM8B** | Transmembrane protein 8B | -0.0567 | 8.93E-03 | 4.85E-01 | 7.07 [7.03-7.11] | 7.01 [6.97-7.05] |
| **MYZAP** | **MYZAP** | Myocardial zonula adherens protein | -0.0650 | 7.04E-05 | 7.33E-02 | 8.69 [8.63-8.76] | 8.63 [8.56-8.69] |
| **RDH12** | **RDH12** | Retinol dehydrogenase 12 | -0.0651 | 6.73E-03 | 4.85E-01 | 8.22 [8.16-8.28] | 8.16 [8.10-8.22] |
| **Caspase-10:region 1** | **CASP10** | Caspase-10:region 1 | -0.0654 | 3.94E-03 | 4.16E-01 | 9.17 [9.07-9.26] | 9.10 [9.00-9.20] |
| **BAMBI:ECD** | **BAMBI** | BMP and activin membrane-bound inhibitor homolog:Extracellular domain | -0.0657 | 4.37E-03 | 4.24E-01 | 7.73 [7.67-7.79] | 7.67 [7.61-7.72] |
| **PKB beta** | **AKT2** | RAC-beta serine/threonine-protein kinase | -0.0659 | 3.81E-03 | 4.09E-01 | 7.55 [7.49-7.61] | 7.49 [7.43-7.55] |
| **Galectin-1** | **LGALS1** | Galectin-1 | -0.0663 | 6.91E-03 | 4.85E-01 | 6.60 [6.55-6.65] | 6.54 [6.49-6.59] |
| **RNF8** | **RNF8** | E3 ubiquitin-protein ligase RNF8 | -0.0667 | 2.56E-03 | 3.52E-01 | 8.82 [8.74-8.90] | 8.75 [8.67-8.83] |
| **sICAM-1** | **ICAM1** | Intercellular adhesion molecule 1 | -0.0684 | 9.44E-03 | 4.85E-01 | 8.46 [8.32-8.60] | 8.39 [8.25-8.53] |
| **FXYD6** | **FXYD6** | FXYD domain-containing ion transport regulator 6 | -0.0697 | 6.14E-03 | 4.85E-01 | 4.10 [4.05-4.16] | 4.03 [3.98-4.09] |
| **UB2R1** | **CDC34** | Ubiquitin-conjugating enzyme E2 R1 | -0.0699 | 1.95E-03 | 3.38E-01 | 6.04 [5.98-6.09] | 5.97 [5.91-6.03] |
| **GIMD1** | **GIMD1** | GTPase IMAP family member GIMD1 | -0.0700 | 7.46E-03 | 4.85E-01 | 7.34 [7.30-7.38] | 7.27 [7.23-7.31] |
| **Beta-dystroglycan** | **DAG1** | Beta-dystroglycan | -0.0706 | 9.14E-03 | 4.85E-01 | 10.92 [10.79-11.04] | 10.84 [10.72-10.97] |
| **K2C71** | **KRT71** | Keratin, type II cytoskeletal 71 | -0.0708 | 7.10E-03 | 4.85E-01 | 7.11 [7.07-7.15] | 7.04 [7.00-7.08] |
| **C3adesArg** | **C3** | C3a anaphylatoxin des Arginine | -0.0714 | 5.09E-05 | 6.18E-02 | 17.56 [17.51-17.61] | 17.49 [17.44-17.54] |
| **INM02** | **EMC10** | UPF0510 protein INM02 | -0.0719 | 7.98E-03 | 4.85E-01 | 9.60 [9.47-9.73] | 9.53 [9.40-9.66] |
| **MP3B2** | **MAP1LC3B2** | Microtubule-associated proteins 1A/1B light chain 3 beta 2 | -0.0733 | 5.02E-03 | 4.42E-01 | 8.39 [8.30-8.47] | 8.31 [8.22-8.40] |
| **CD79A** | **CD79A** | B-cell antigen receptor complex-associated protein alpha chain | -0.0736 | 4.78E-05 | 6.18E-02 | 8.06 [8.02-8.09] | 7.98 [7.94-8.02] |
| **LRC52** | **LRRC52** | Leucine-rich repeat-containing protein 52 | -0.0740 | 2.64E-03 | 3.53E-01 | 6.79 [6.74-6.84] | 6.72 [6.67-6.77] |
| **KIF3A** | **KIF3A** | Kinesin-like protein KIF3A | -0.0773 | 7.12E-03 | 4.85E-01 | 8.70 [8.65-8.76] | 8.63 [8.57-8.68] |
| **MUC1:region 2** | **MUC1** | Mucin-1:region 2 | -0.0775 | 8.55E-03 | 4.85E-01 | 8.69 [8.63-8.76] | 8.62 [8.55-8.69] |
| **Sorcin** | **SRI** | Sorcin | -0.0780 | 9.87E-03 | 4.93E-01 | 5.12 [5.05-5.19] | 5.04 [4.97-5.11] |
| **LY86** | **LY86** | Lymphocyte antigen 86 | -0.0780 | 6.14E-03 | 4.85E-01 | 11.92 [11.83-12.02] | 11.85 [11.75-11.94] |
| **RAB38** | **RAB38** | Ras-related protein Rab-38 | -0.0783 | 1.03E-03 | 2.68E-01 | 6.82 [6.75-6.89] | 6.74 [6.67-6.81] |
| **EYA2** | **EYA2** | Eyes absent homolog 2 | -0.0784 | 3.78E-03 | 4.09E-01 | 7.81 [7.67-7.95] | 7.73 [7.59-7.87] |
| **CKLF4** | **CMTM4** | CKLF-like MARVEL transmembrane domain-containing protein 4 | -0.0784 | 1.28E-03 | 2.77E-01 | 8.27 [8.19-8.35] | 8.19 [8.11-8.27] |
| **MCP-1** | **CCL2** | C-C motif chemokine 2 | -0.0786 | 8.99E-03 | 4.85E-01 | 7.76 [7.68-7.84] | 7.68 [7.60-7.76] |
| **MMP-8** | **MMP8** | Neutrophil collagenase | -0.0786 | 8.63E-03 | 4.85E-01 | 10.01 [9.87-10.15] | 9.93 [9.79-10.07] |
| **FUT9** | **FUT9** | Alpha-(1,3)-fucosyltransferase 9 | -0.0797 | 4.26E-04 | 2.07E-01 | 8.16 [8.13-8.20] | 8.08 [8.05-8.12] |
| **CK5P3** | **CDK5RAP3** | CDK5 regulatory subunit-associated protein 3 | -0.0817 | 3.15E-03 | 3.89E-01 | 9.32 [9.24-9.40] | 9.24 [9.15-9.32] |
| **F110A** | **FAM110A** | Protein FAM110A | -0.0824 | 7.59E-04 | 2.38E-01 | 7.02 [6.93-7.11] | 6.94 [6.85-7.03] |
| **CETN2** | **CETN2** | Centrin-2 | -0.0827 | 7.28E-03 | 4.85E-01 | 8.48 [8.42-8.53] | 8.39 [8.34-8.45] |
| **ASCC1** | **ASCC1** | Activating signal cointegrator 1 complex subunit 1 | -0.0830 | 6.56E-03 | 4.85E-01 | 7.22 [7.16-7.28] | 7.13 [7.07-7.19] |
| **CDK15** | **CDK15** | Cyclin-dependent kinase 15; EC=2.7.11.22 | -0.0846 | 3.56E-03 | 4.09E-01 | 10.07 [10.02-10.11] | 9.98 [9.93-10.03] |
| **CN37** | **CNP** | 2',3'-cyclic-nucleotide 3'-phosphodiesterase | -0.0852 | 7.22E-03 | 4.85E-01 | 9.59 [9.45-9.73] | 9.50 [9.36-9.64] |
| **GUAD** | **GDA** | Guanine deaminase | -0.0856 | 6.53E-04 | 2.27E-01 | 8.29 [8.23-8.35] | 8.20 [8.15-8.26] |
| **AIF1** | **AIF1** | Allograft inflammatory factor 1 | -0.0888 | 7.69E-03 | 4.85E-01 | 11.13 [11.03-11.23] | 11.04 [10.94-11.15] |
| **GD1L1** | **GDAP1L1** | Ganglioside-induced differentiation-associated protein 1-like 1 | -0.0896 | 7.24E-04 | 2.38E-01 | 8.82 [8.74-8.91] | 8.73 [8.65-8.82] |
| **ATF6B** | **ATF6B** | Cyclic AMP-dependent transcription factor ATF-6 beta | -0.0912 | 9.52E-03 | 4.85E-01 | 8.70 [8.58-8.82] | 8.61 [8.49-8.73] |
| **DC-SIGNR** | **CLEC4M** | C-type lectin domain family 4 member M | -0.0913 | 1.31E-03 | 2.77E-01 | 12.08 [12.01-12.14] | 11.98 [11.92-12.05] |
| **HN1** | **JPT1** | Hematological and neurological expressed 1 protein | -0.0916 | 6.43E-03 | 4.85E-01 | 7.35 [7.11-7.59] | 7.26 [7.01-7.51] |
| **DDHD2** | **DDHD2** | Phospholipase DDHD2 | -0.0921 | 8.48E-03 | 4.85E-01 | 7.82 [7.72-7.92] | 7.73 [7.63-7.83] |
| **CASC4** | **GOLM2** | Protein CASC4 | -0.0924 | 2.48E-03 | 3.52E-01 | 9.09 [8.97-9.21] | 9.00 [8.88-9.12] |
| **S100P** | **S100P** | Protein S100-P | -0.0928 | 6.01E-03 | 4.85E-01 | 7.24 [7.18-7.29] | 7.14 [7.09-7.20] |
| **RHOG** | **RHOG** | Rho-related GTP-binding protein RhoG | -0.0929 | 2.71E-03 | 3.53E-01 | 8.21 [8.14-8.27] | 8.11 [8.05-8.18] |
| **TXNL1** | **TXNL1** | Thioredoxin-like protein 1 | -0.0931 | 4.54E-03 | 4.24E-01 | 7.44 [7.35-7.54] | 7.35 [7.25-7.45] |
| **IL-34** | **IL34** | Interleukin-34 | -0.0937 | 7.15E-06 | 1.30E-02 | 9.16 [9.07-9.25] | 9.07 [8.97-9.16] |
| **MED10** | **MED10** | Mediator of RNA polymerase II transcription subunit 10 | -0.0940 | 3.82E-03 | 4.09E-01 | 8.23 [8.16-8.29] | 8.13 [8.06-8.20] |
| **Tpo** | **THPO** | Thrombopoietin | -0.0951 | 7.25E-03 | 4.85E-01 | 6.43 [6.38-6.49] | 6.34 [6.28-6.39] |
| **FKBP6** | **FKBP6** | Inactive peptidyl-prolyl cis-trans isomerase FKBP6 | -0.0952 | 5.74E-04 | 2.09E-01 | 8.27 [8.23-8.32] | 8.18 [8.13-8.22] |
| **KRIP-1** | **TRIM28** | Transcription intermediary factor 1-beta | -0.0955 | 2.45E-03 | 3.52E-01 | 7.99 [7.88-8.10] | 7.89 [7.78-8.00] |
| **Cytochrome P450 3A4** | **CYP3A4** | Cytochrome P450 3A4 | -0.0982 | 8.80E-04 | 2.46E-01 | 10.71 [10.62-10.79] | 10.61 [10.52-10.70] |
| **CLC4G** | **CLEC4G** | C-type lectin domain family 4 member G | -0.1006 | 4.52E-03 | 4.24E-01 | 10.13 [9.72-10.54] | 10.03 [9.62-10.45] |
| **WISP-1** | **CCN4** | WNT1-inducible-signaling pathway protein 1 | -0.1014 | 2.71E-04 | 1.98E-01 | 7.98 [7.85-8.12] | 7.88 [7.74-8.02] |
| **DPEP1** | **DPEP1** | Dipeptidase 1 | -0.1016 | 8.70E-03 | 4.85E-01 | 7.42 [7.36-7.48] | 7.32 [7.26-7.38] |
| **C3** | **C3** | Complement C3 | -0.1030 | 1.11E-03 | 2.69E-01 | 17.24 [17.15-17.33] | 17.14 [17.05-17.23] |
| **FTMT** | **FTMT** | Ferritin, mitochondrial | -0.1032 | 2.38E-03 | 3.52E-01 | 12.56 [12.48-12.63] | 12.45 [12.38-12.53] |
| **BSP** | **IBSP** | Bone sialoprotein 2 | -0.1035 | 2.50E-03 | 3.52E-01 | 7.74 [7.62-7.86] | 7.63 [7.51-7.75] |
| **SPC1L** | **SPATC1L** | Speriolin-like protein | -0.1051 | 2.17E-03 | 3.51E-01 | 8.84 [8.73-8.94] | 8.73 [8.63-8.84] |
| **Cardiotrophin-1** | **CTF1** | Cardiotrophin-1 | -0.1060 | 8.41E-03 | 4.85E-01 | 9.30 [9.04-9.56] | 9.20 [8.93-9.46] |
| **NSF** | **NSF** | Vesicle-fusing ATPase | -0.1084 | 4.81E-03 | 4.38E-01 | 9.10 [8.96-9.24] | 8.99 [8.85-9.13] |
| **Testican-2** | **SPOCK2** | Testican-2 | -0.1093 | 1.49E-03 | 2.86E-01 | 15.33 [15.19-15.46] | 15.22 [15.08-15.35] |
| **CD133** | **PROM1** | CD133 antigen | -0.1165 | 3.77E-03 | 4.09E-01 | 8.87 [8.81-8.93] | 8.75 [8.69-8.81] |
| **FBXL4:LRR4 and LRR5** | **FBXL4** | F-box/LRR-repeat protein 4:Leucine-rich repeats 4 and 5 | -0.1178 | 5.54E-04 | 2.09E-01 | 7.69 [7.62-7.76] | 7.57 [7.50-7.64] |
| **Aconitase 1** | **ACO1** | Cytoplasmic aconitate hydratase | -0.1179 | 6.93E-03 | 4.85E-01 | 7.37 [7.29-7.46] | 7.26 [7.17-7.34] |
| **B3GT5** | **B3GALT5** | Beta-1,3-galactosyltransferase 5 | -0.1193 | 4.45E-03 | 4.24E-01 | 9.13 [9.00-9.25] | 9.01 [8.88-9.13] |
| **PP4C** | **PPP4C** | Serine/threonine-protein phosphatase 4 catalytic subunit | -0.1222 | 7.83E-04 | 2.38E-01 | 10.17 [10.07-10.27] | 10.05 [9.94-10.15] |
| **GPR110** | **ADGRF1** | Adhesion G-protein coupled receptor F1 | -0.1230 | 3.70E-03 | 4.09E-01 | 9.61 [9.50-9.73] | 9.49 [9.38-9.61] |
| **APBB3:PID 2** | **APBB3** | Amyloid beta A4 precursor protein-binding family B member 3:Phosphotyrosine Interaction Domain 2, Isoform IV | -0.1231 | 5.26E-04 | 2.09E-01 | 8.21 [8.13-8.28] | 8.08 [8.01-8.16] |
| **TM59L** | **TMEM59L** | Transmembrane protein 59-like | -0.1235 | 8.73E-03 | 4.85E-01 | 9.87 [9.67-10.07] | 9.74 [9.54-9.95] |
| **ITPK1** | **ITPK1** | Inositol-tetrakisphosphate 1-kinase | -0.1238 | 2.49E-03 | 3.52E-01 | 9.88 [9.71-10.05] | 9.76 [9.58-9.93] |
| **AT5F1** | **ATP5PB** | ATP synthase B chain, mitochondrial | -0.1255 | 3.01E-03 | 3.78E-01 | 9.19 [9.11-9.27] | 9.07 [8.99-9.15] |
| **HLA-G** | **HLA-G** | HLA class I histocompatibility antigen, alpha chain G | -0.1262 | 9.44E-03 | 4.85E-01 | 13.35 [13.18-13.51] | 13.22 [13.06-13.39] |
| **STK10** | **STK10** | Serine/threonine kinase 10 | -0.1275 | 3.24E-03 | 3.94E-01 | 8.60 [8.47-8.74] | 8.48 [8.34-8.61] |
| **NPY** | **NPY** | Neuropeptide Y | -0.1296 | 9.01E-03 | 4.85E-01 | 10.01 [9.88-10.14] | 9.88 [9.75-10.01] |
| **GPR56** | **ADGRG1** | Adhesion G-protein coupled receptor G1 | -0.1310 | 9.58E-03 | 4.85E-01 | 8.54 [8.38-8.69] | 8.40 [8.25-8.56] |
| **SCF** | **KITLG** | Kit ligand | -0.1335 | 5.10E-03 | 4.42E-01 | 9.51 [9.37-9.65] | 9.38 [9.23-9.52] |
| **iC3b** | **C3** | Complement C3b, inactivated | -0.1424 | 1.92E-03 | 3.38E-01 | 16.37 [16.27-16.48] | 16.23 [16.12-16.34] |
| **SETMR** | **SETMAR** | Histone-lysine N-methyltransferase SETMAR | -0.1454 | 1.33E-03 | 2.77E-01 | 7.91 [7.85-7.98] | 7.77 [7.70-7.83] |
| **GOT1** | **GOT1** | Aspartate aminotransferase, cytoplasmic | -0.1456 | 3.63E-04 | 2.07E-01 | 7.99 [7.88-8.10] | 7.84 [7.73-7.96] |
| **MACD2** | **MACROD2** | O-acetyl-ADP-ribose deacetylase MACROD2 | -0.1487 | 8.96E-04 | 2.46E-01 | 7.21 [7.12-7.29] | 7.06 [6.97-7.15] |
| **ZCC18** | **ZCCHC18** | Zinc finger CCHC domain-containing protein 18 | -0.1505 | 1.16E-03 | 2.69E-01 | 8.61 [8.53-8.69] | 8.46 [8.38-8.54] |
| **UNG** | **UNG** | Uracil-DNA glycosylase | -0.1556 | 9.11E-04 | 2.46E-01 | 8.18 [8.02-8.34] | 8.02 [7.86-8.19] |
| **Mammaglobin 2** | **SCGB2A1** | Mammaglobin-B | -0.1627 | 9.07E-03 | 4.85E-01 | 10.03 [9.93-10.13] | 9.87 [9.76-9.97] |
| **AP1AR** | **AP1AR** | AP-1 complex-associated regulatory protein | -0.1759 | 6.17E-03 | 4.85E-01 | 9.74 [9.51-9.96] | 9.56 [9.33-9.79] |
| **Endothelial lipase** | **LIPG** | Endothelial cell-derived lipase | -0.1822 | 5.43E-04 | 2.09E-01 | 7.11 [6.97-7.25] | 6.93 [6.78-7.07] |
| **Dermokine** | **DMKN** | Dermokine | -0.2049 | 7.13E-03 | 4.85E-01 | 9.30 [9.17-9.44] | 9.10 [8.96-9.23] |
| **40S ribosomal protein SA** | **RPSA** | 40S ribosomal protein SA | -0.2298 | 1.12E-03 | 2.69E-01 | 8.26 [8.15-8.36] | 8.03 [7.92-8.14] |
| **THOP1** | **THOP1** | Thimet oligopeptidase | -0.2328 | 1.74E-04 | 1.41E-01 | 7.11 [7.03-7.20] | 6.88 [6.79-6.96] |
| **FGL1** | **FGL1** | Fibrinogen-like protein 1 | -0.2482 | 8.17E-03 | 4.85E-01 | 6.93 [6.73-7.12] | 6.68 [6.48-6.88] |
| **IL-18 Rb** | **IL18RAP** | Interleukin-18 receptor accessory protein | -0.2653 | 6.90E-03 | 4.85E-01 | 7.45 [7.25-7.65] | 7.18 [6.98-7.39] |
| **C3d** | **C3** | Complement C3d fragment | -0.3116 | 1.80E-03 | 3.28E-01 | 14.59 [14.32-14.86] | 14.28 [14.00-14.55] |
| **LDHA** | **LDHA** | L-lactate dehydrogenase A chain | -0.3170 | 3.38E-04 | 2.07E-01 | 9.61 [9.41-9.81] | 9.30 [9.09-9.50] |
| **K0494:C-term** | **EFCAB14** | EF-hand calcium-binding domain-containing protein 14:C-term | -0.3279 | 3.85E-04 | 2.07E-01 | 7.87 [7.72-8.03] | 7.55 [7.39-7.71] |
| **C3a** | **C3** | C3a anaphylatoxin | -0.6068 | 1.70E-03 | 3.18E-01 | 12.73 [12.33-13.12] | 12.12 [11.72-12.52] |
