# Supplementary material for: Efficacy of intrathecal mesenchymal stem cell-neural progenitor therapy in progressive MS: results from a phase II, randomized, placebo-controlled clinical trial
Source: Stem Cell Res Ther. 2024 May 23;15:151. doi: 10.1186/s13287-024-03765-6 (PMC11119709; doi:10.1186/s13287-024-03765-6)
Supplement: Supplementary file 4 — Supplementary Material 4 [file 13287_2024_3765_MOESM4_ESM.docx]

**
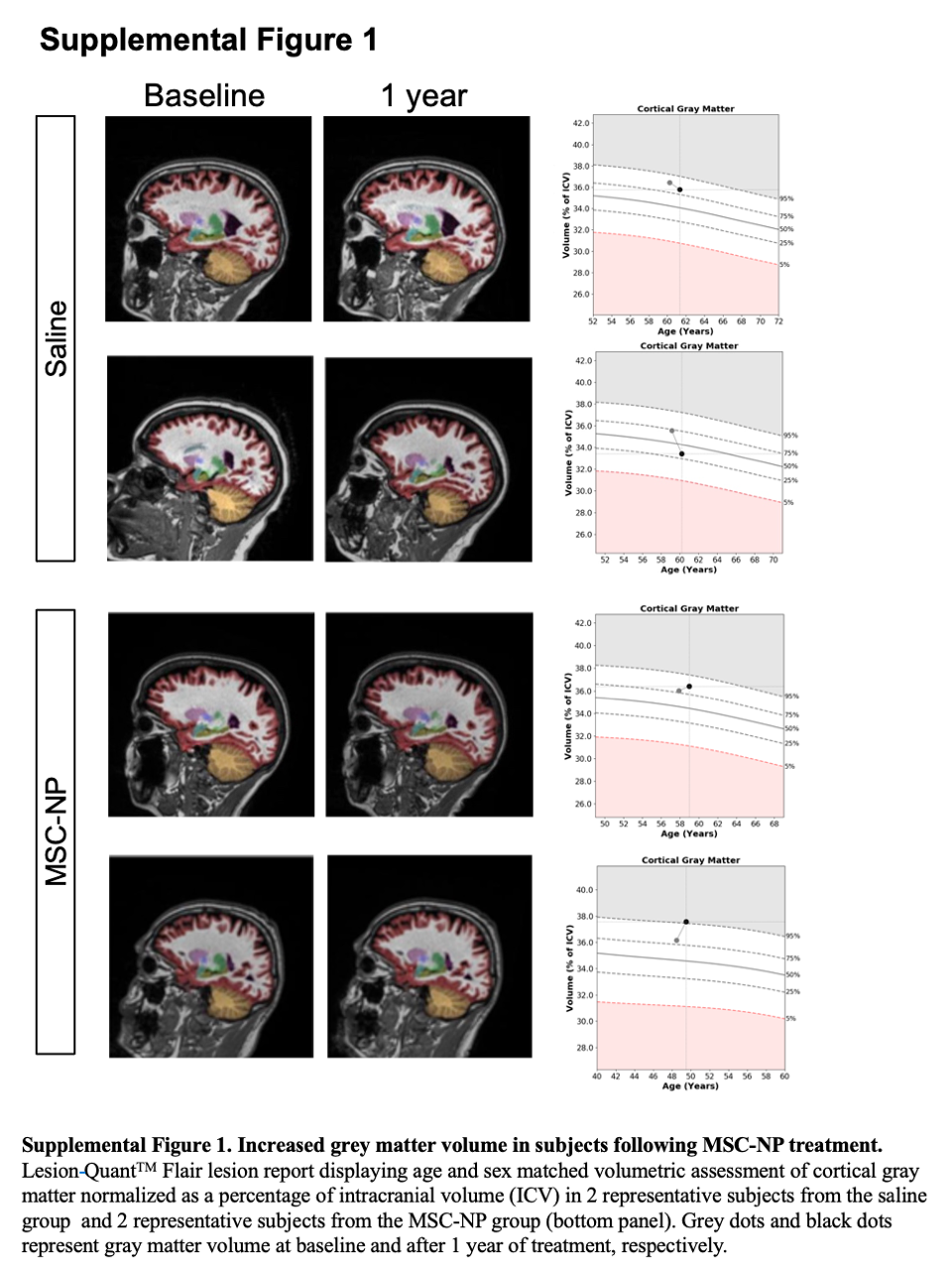
Supplemental Figure 1**

**Supplemental Figure 1. Increased grey matter volume in subjects following MSC-NP treatment.** Lesion Quant^TM^ Flair lesion report results in 2 representative subjects from the saline group (top 2 panels) and 2 representative subjects from the MSC-NP group (bottom 2 panels). Representative MRI images demonstrating grey matter volume (red shading) at baseline and after 1 year of treatment with either saline or MSC-NPs. Graphs depict age-adjusted cortical grey matter volume as a % intracranial volume (%ICV). Grey dots and black dots represent gray matter volume at baseline and after 1 year of treatment, respectively.
